# Structural basis of the AlgU-MucA^cyto^ interaction and SspB-mediated degradation in *Pseudomonas aeruginosa* stress response

**DOI:** 10.1128/mbio.00148-26

**Published:** 2026-05-19

**Authors:** Tao Li, Yingzhi Wang, Ninglin Zhao, Chunlei Ge, Cuiling Wu, Ke Li, Li Li, Zhiqiang Wang, Ying Chen, Zhenpu Chen, Weike Li, Yang Liu, Zhonghui Wang, Yun Sha, Hong Yao, Yibo Zhu, Rui Bao

**Affiliations:** 1 Cancer Biotherapy Center & Cancer Research Institute, Yunnan Cancer Hospital, The Third Affiliated Hospital of Kunming Medical University, Peking University Cancer Hospital Yunnan 531840 , Kunming, China; 2 University-Town Hospital of Chongqing Medical University 568864, Chongqing, China; 3 Center of Infectious Diseases, Division of Infectious Diseases in State Key Laboratory of Biotherapy, West China Hospital, Sichuan University 34753, Chengdu, Sichuan, China; 4 Department of Radiation Oncology, First Affiliated Hospital of Kunming Medical University 36657, Kunming, Yunnan, China; 5 Accurate Biotechnology (Hunan) Co., Ltd., Changsha, China; Florida International University, Miami, Florida, USA

**Keywords:** *Pseudomonas aeruginosa*, σ factor AlgU, MucA-RIP, alginate, virulence factors, SspB adaptor, ClpX/ClpP protease

## Abstract

In *Pseudomonas aeruginosa*, the sigma factor AlgU functions as a critical stress-responsive regulator governing essential physiological processes. This study assessed *algU*-dependent phenotypes through targeted gene knockout, including effects on alginate secretion and host cell invasion. Furthermore, an innovative fusion protein strategy addressed complex preparation challenges, enabling the determination of a high-resolution (2.101 Å) structure of the AlgU-MucA^cyto^ complex and revealing an exceptionally low backbone deviation of merely 0.01 Å relative to the reported 6IN7 complex. Structural analysis revealed an extensive mutation-resistant interaction network at the binding interface, stabilized primarily by salt bridges, hydrogen bonds, and hydrophobic interactions. Subsequent mutational validation confirmed that triple or sextuple alanine substitutions at critical interaction sites maintained complex binding integrity while significantly compromising thermal stability, indicating that although dispensable for binding, these residues are indispensable for complex stability. Crucially, pull-down assays identified SspB as the essential adaptor bridging the AlgU-MucA^cyto^ complex to the ClpX/ClpP protease; AlphaFold3 modeling and quantitative BLI measurements reveal that SspB engages AlgU via its N-terminal domain and binds ClpX through its disordered C-terminus, enabling substrate delivery and proteolysis-coupled AlgU release. Collectively, these findings elucidate a dual-safeguard regulatory mechanism: inherent mutation resistance at the binding interface prevents aberrant AlgU activation by common clinical *mucA* mutations, while SspB-mediated proteolysis ensures stimulus-responsive AlgU liberation. This evolutionary adaptation confers resistance against detrimental AlgU overactivation in *P. aeruginosa* and provides novel therapeutic targets.

**IMPORTANCE:**

Deletion of the *algU* gene affected the production of pyoverdine, pyocyanin, and alginate in *Pseudomonas aeruginosa*, as well as its cell invasiveness. By fusing AlgU and MucA^cyto^, we were able to obtain an AlgU-MucA^cyto^ protein complex with both high yield and purity, and successfully obtained a crystal structure of the AlgU-MucA^cyto^ complex. Structural analysis revealed an extensive and robust network of salt bridges, hydrogen bonds, and charge interactions between the two proteins. Multi-site alanine substitutions (up to sextuple mutations) in interfacial charged clusters of the AlgU-MucA^cyto^ complex fail to disrupt binding but severely impair thermal stability, revealing that these residues are binding-redundant yet stability-essential. Structural modeling and BLI measurements identify SspB as a dual-interface adaptor: its N-terminal domain recognizes the preassembled AlgU-MucA^cyto^ complex (via the AlgU hairpin surface), whereas its flexible C-terminal tail engages ClpX, thereby bridging AlgU-MucA^cyto^ to ClpXP and positioning MucA^cyto^ for proteolysis-coupled AlgU release.

## INTRODUCTION

*Pseudomonas aeruginosa* (*P. aeruginosa*) is a common opportunistic pathogen and one of the leading causes of hospital-acquired infections. It is known for its high phenotypic diversity and adaptability, largely due to its relatively larger genome (1, 2) and a significant proportion of regulatory genes (1, 3). These regulatory genes are integral to various physiological processes, including cell adhesion, mucoid, biofilm formation, antibiotic resistance, and virulence factor secretion (4, 5), thereby enabling *P. aeruginosa* to adapt quickly to changing environments. Research has also identified *P. aeruginosa* as a primary bacterial co-infection contributing to patient fatalities during the COVID-19 pandemic, with its high virulence and antibiotic resistance posing a significant threat to critically ill patients (6).

AlgU, an extracellular function sigma factor (σ^ECF^), is essential for stress signal transduction and amplification within the envelope damage stress response (ESRs) system. It plays a critical role in the regulation of multiple physiological processes in *P. aeruginosa*, contributing significantly to its adaptability and acquired antibiotic resistance. Notably, *E. coli* σ^E^ (RpoE) and *P. aeruginosa* AlgU are functionally interchangeable, supporting the evolutionary conservation of σ^E^/AlgU-centered envelope stress regulatory modules (7). Under non-induced conditions, AlgU is regulated by the anti-sigma factor MucA and the intracellular negative regulator MucB, forming a “spindle-shaped” complex (8). The activation of AlgU involves a cascade of proteolysis by three proteases—AlgW, MucP, and ClpX/ClpP. These proteinases target different regions of MucA, a process known as MucA-RIP. Our previous research has elucidated the structures of key proteins (MucA-MucB, AlgW) involved in the MucA-RIP pathway (9, 10), and the lipids/peptides signal-dependent activation mechanism, along with its effects on alginate synthesis, virulence factor secretion, mucoid phenotype transition, and biofilm formation in *P. aeruginosa*. Clinical isolates frequently harbor mucA mutations (e.g., *mucA*22) that inactivate the anti-σ factor MucA (11, 12), resulting in constitutive AlgU activation and increased alginate production that manifests as a mucoid phenotype (13, 14). Interestingly, *algU* inactivation has also been reported to increase systemic virulence in acute mouse infection models, despite sensitizing cells to reactive oxygen intermediates and phagocyte-mediated killing, highlighting the context-dependent roles of AlgU during infection (15).

Nevertheless, the AlgU-MucA^cyto^ complex, a key negative regulatory product in the MucA-RIP pathway, remains combined even after detachment from the membrane, significantly inhibiting AlgU activation and its regulation of downstream genes (9, 10, 16). The AlgU-MucA^cyto^ complex is then delivered to the ClpX/ClpP protease complex for further proteolysis of MucA, which ultimately determines the final activation of AlgU. Although the crystal structure of the AlgU-MucA^cyto^ complex has been determined (17), the mechanism by which the intracellular AlgU-MucA^cyto^ complex is specifically recognized by an adaptor protein and recruited to the ClpX/ClpP protease complex for further degradation of MucA^cyto^ remains unclear.

In this study, we utilized gene knockout technology to confirm the biological activity of *algU* in regulating the secretion of virulence factors and the mucoid phenotype in *P. aeruginosa*. Furthermore, we reported the crystal structure of the AlgU-MucA^cyto^ complex through a fusion protein expression model. Structural analysis revealed that an extensive and robust network of interactions, including salt bridges, hydrogen bonds, and charge-based interactions, exists between the cytoplasmic domain of MucA^cyto^ and AlgU. Crucially, localized mutations or single-point mutations are insufficient to completely disrupt the binding between MucA^cyto^ and AlgU. This binding stability prevents the detrimental consequences of overactivation, namely unnecessary stress responses and cell death. This property reflects an evolutionary advantage in *P. aeruginosa*. Furthermore, we confirmed that the SspB protein can interact independently with both the (AlgU-MucA^cyto^) complex and the ClpX protein. This demonstrates that SspB is the key adaptor protein responsible for mediating the recruitment of the AlgU-MucA^cyto^ complex to the ClpX/ClpP protease complex, facilitating the subsequent degradation of the MucA^cyto^. This finding holds significant importance for an in-depth understanding of the unique functions of AlgU and the activation mechanism of stress-responsive sigma factors.

## RESULTS

### AlgU is an important σ^ECF^ in many physiological processes of *P. aeruginosa*

**Fig 1:**
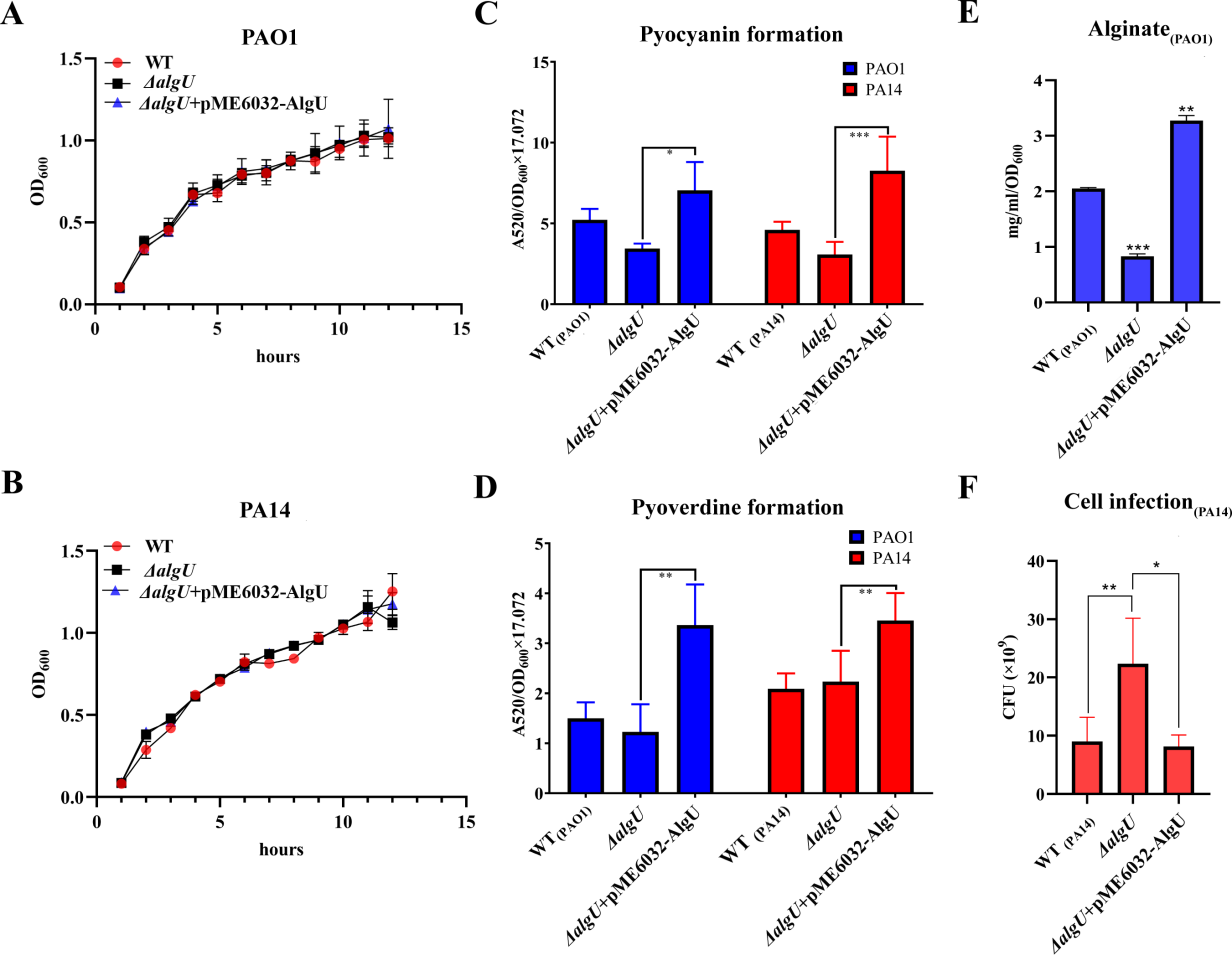
Disruption of the *algU* gene in *P. aeruginosa* affects its metabolic product generation and cell invasive ability. The algU gene knockout does not hamper bacterial growth in PAO1 (**A**) and PA14 (**B**) strains. The effect of *algU* on pyocyanin (**C**), pyoverdine (**D**) formation, alginate (**E**) secretion phenotype, and cell infection (**F**) was tested on PAO1 or PA14 strains. A rescue experiment was conducted by expressing *algU* in the Δ*algU* strain using the pME6032 plasmid. One-way ANOVA statistical test with equal variances was conducted. *, *P* < 0.05; **, *P* < 0.01; ***, *P* < 0.001. Original data for alginate determination and CFU counting are in Fig. S1.

Utilizing a two-step allelic exchange strategy, the *algU* gene was successfully disrupted in both the wild-type (WT) non-mucoid PAO1 and PA14 strains of *P. aeruginosa*. The resulting knockout strains, denoted as Δ*algU*, were confirmed through PCR and sequencing and found viable (Fig. S1A). To complement the *algU* deletion, the multicopy plasmid pME6032-AlgU driven by the strong, IPTG-inducible P_Tac_ promoter was introduced into the Δ*algU* strain. Growth curves were established and revealed that the knockout did not affect the growth rates of PAO1 and PA14 strains at 37°C (Fig. 1A and B). Upon analyzing the metabolite products, it was observed that the Δ*algU* strain showed a slight, yet statistically insignificant, decrease in pyocyanin production. In contrast, the complemented strains exhibited an increase in pyocyanin production that was statistically significant when compared to the Δ*algU* strains (Fig. 1C). No change in pyoverdine levels was detected between the wild-type and Δ*algU* strains. However, the complemented strain displayed a significant increase compared to the Δ*algU* strain (Fig. 1D). Notably, compared with the PAO1 strain, alginate production in the Δ*algU* strain was found to be significantly reduced by approximately 50%. When the Δ*algU* strain was complemented, alginate production increased relative to the Δ*algU* strain and exceeded the wild-type PAO1 level under these induction conditions (Fig. 1E; Fig. S1B and C). Additionally, the cell invasion ability of the PA14 Δ*algU* strain was evaluated. Surprisingly, contrary to expectations of diminished cell-invasive capabilities, the Δ*algU* strain exhibited a significant enhancement in cell-invasive abilities. The complemented strain displayed a decrease in cell invasion capabilities, consistent with the level observed in the WT strain and showing a notable difference compared to the Δ*algU* strain (Fig. 1F; Fig. S1D). Taken together, these assays provide contextual phenotyping consistent with AlgU influencing alginate output and host-cell interaction phenotypes in *P. aeruginosa*.

### Overcoming low-yield expression via fusion construct enables high-resolution structure of the AlgU-MucA^cyto^ complex

**Fig 2:**
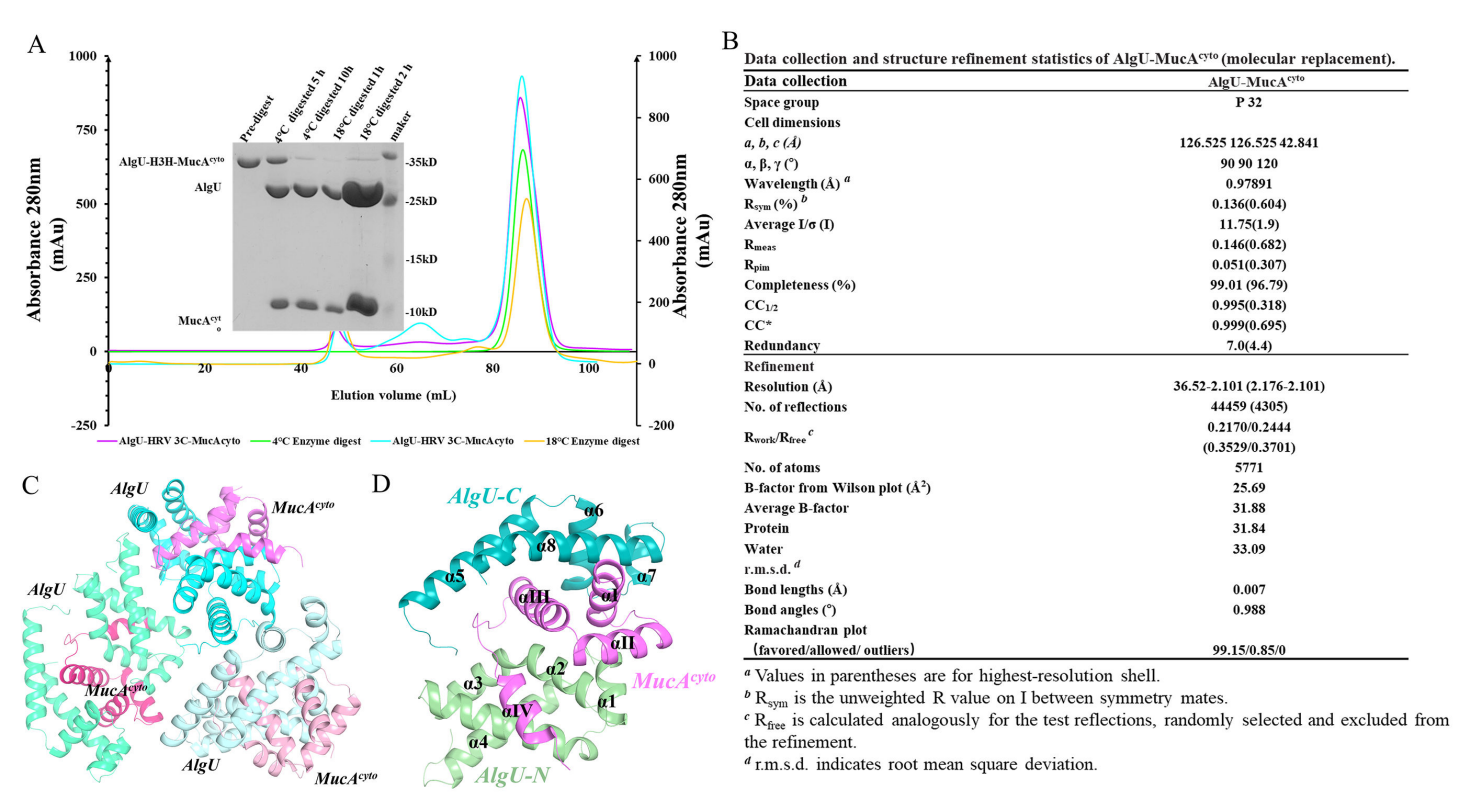
AlgU-MucA^cyto^ complex preparation and structural analysis. (**A**) The AlgU-MucA^cyto^ complex was generated through HRV 3C protease cleavage of the AlgU-H3H-MucA^cyto^ fusion protein. (**B**) Crystallographic data collection and refinement statistics for the AlgU-MucA^cyto^ complex structure. (**C**) The AlgU-MucA^cyto^ complex forms a trimer with a space group of P32. (**D**) Overall architecture of the AlgU-MucA^cyto^ monomeric unit.

In the MucA-RIP pathway, upon exposure to external stimuli, the AlgU and MucA^cyto^ complex dissociates from the cell membrane. However, AlgU and MucA^cyto^ remain tightly bound to each other, which affects the subsequent regulatory functions of AlgU. To address this, we have investigated the interaction between AlgU and MucA^cyto^ using crystallographic methods. First, the purification of AlgU and MucA^cyto^ (1–80, the core cytosolic region bound by AlgU [17]) individually failed to yield a large quantity of high-quality protein, so we adopted a fusion protein to obtain the AlgU-MucA^cyto^ complex, rather than fully recapitulating the *in vivo* RIP cascade process or modeling mucoidy *in vivo*. By using a linker containing an HRV 3C protease cleavage site (LEVLFQ↓GP) and His_6_ tag (denoted H3H tag), the AlgU and MucA^cyto^ proteins were fused together, resulting in a high-yield and purified AlgU-H3H-MucA^cyto^ fusion protein (Fig. S2A). Subsequent protease digestion experiments demonstrated that while HRV 3C protease cleaved AlgU-H3H-MucAcyto into the individual proteins AlgU and MucA^cyto^ (Fig. 2A), the pre- and post-digestion protein populations co-eluted with identical chromatographic profiles on size-exclusion chromatography (SEC) (Fig. S3). This indicates that AlgU and MucA^cyto^ retain strong mutual binding, forming a stable complex. This complex was subsequently purified by gel-filtration and yielded a crystal that diffracted to 2.101 Å (Fig. S4A and B). The *R*_free_ and *R*_work_ of final refined structure is 0.244 and 0.217, respectively (Fig. 2B). The AlgU-MucA^cyto^ complex forms a trimer with a space group of P32 (Fig. 2C). Overall structure of the AlgU-MucA^cyto^ complex is composed of 12 alpha helices (Fig. 2D). The N-terminal domain (NTD; AlgU-N) and C-terminal domain (CTD; AlgU-C) of AlgU are each composed of four alpha helices, forming a hairpin-like structure that encapsulates α1 and α3 of MucA^cyto^ (Fig. 2D). Additionally, the α2 of MucA^cyto^ forms tight interactions with α1 in AlgU-N and α7 in AlgU-C, effectively locking the hairpin’s opening and allowing AlgU to better encapsulate α1 and α3 in MucA^cyto^. The interactions between α4 of MucA^cyto^ and α2 as well as α3 in AlgU-N domain are weaker and may serve a stabilizing role for the overall structure (Fig. 2D).

Furthermore, we compared the well-defined AlgU-MucA^cyto^ complex with the previously established structure (PDB code 6IN7 [17]) and found that the root-mean-square deviation (RMSD) measured across 253 C-α atoms was 0.01 Å (Fig. S5A). When comparing with the functional mode of RopE-RseA^cyto^ in *Escherichia coli* (*E. coli*), a superposition of the two structures reveals a similar conformation (Fig. S5B). The RMSD between them is 2.529 Å, as measured across 156 C-α atoms, indicating that despite being from different species, these protein complexes share a relatively consistent three-dimensional arrangement. This structural homology could imply analogous mechanisms of action or interactions within their respective cellular contexts.

### Structural basis for high-affinity AlgU-MucA^cyto^ binding via charged and polar networks

**Fig 3:**
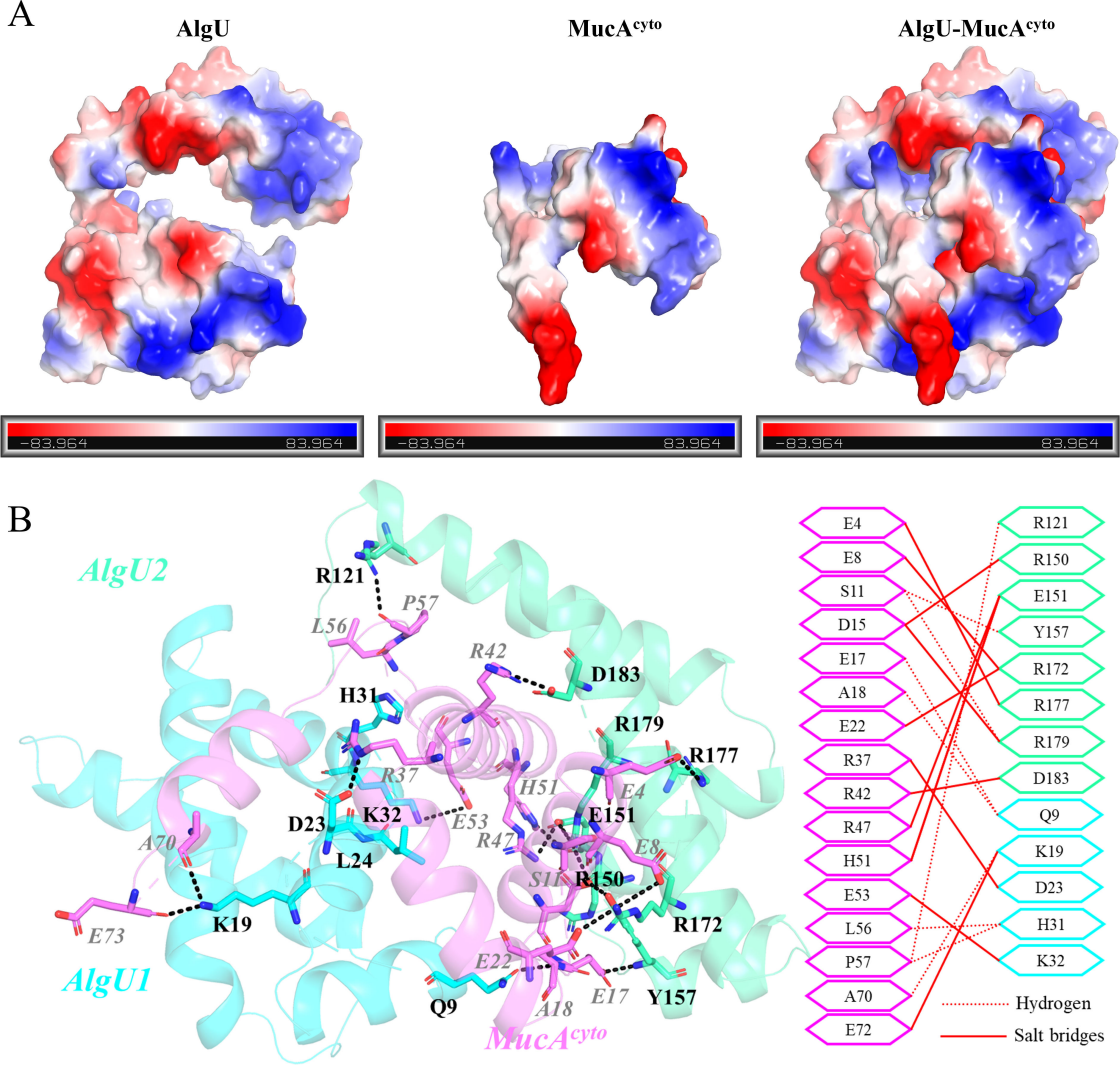
Charge distribution and residue interaction in complex AlgU-MucA^cyto^. (**A**) The surface charge distribution on the complex of AlgU-MucA^cyto^ is depicted, with negatively charged regions indicated in red and positively charged regions in blue. (**B**) The structures and schematics illustrate the binding interfaces between AlgU and MucA^cyto^ as depicted in the left panel, with its NTD in cyan and CTD in light green, and MucA^cyto^, which is depicted in purple. The hydrogen and salt bridge interactions are summarized in the right panel.

Examining the structure of the AlgU-MucA^cyto^ complex, it becomes evident that their interaction occurs over a relatively broad buried interface area of 2,976 Å (2) with a solvation free energy gain (ΔiG) of −28.6 kcal/mol (measured by PDBePISA: https://www.ebi.ac.uk/pdbe/pisa/). Further investigation of the surface charge distribution in the AlgU-MucA^cyto^ complex structure shows a perfect match on the surface charge distributions of AlgU and MucA^cyto^, which means that the binding stability of AlgU and MucA^cyto^ is driven and maintained primarily by charge–charge interactions (Fig. 3A).

Upon closer analysis of the structure of the AlgU-MucA^cyto^ complex, about 36.7% (62/169) of the residues in AlgU and about 79.2% (57/72) residues in MucA^cyto^ were involved in interactions, and many interaction sites were well identified, among which were particularly prominent and dominated by extensive hydrogen bonding and salt bridge interaction networks, such as E4, E8, E17, E22, R37, R42, R47, E53, and E72 in MucA^cyto^ and K19, D23, K32, E151, R172, R179, and D183 in AlgU (Fig. 3B), the distance between these key residues is less than 3 Å. More prevalent hydrophobic amino acid interactions are shown in Fig. S6. This comprehensive structural analysis reveals that the exceptional stability of the AlgU-MucA^cyto^ complex is governed by complementary electrostatic matching across an extensive interface, reinforced by dense hydrogen-bonding/salt-bridge networks involving >35% of residues, with hydrophobic packing providing additional stabilization.

### Critical residues in the AlgU-MucA^cyto^ complex sustain stability despite binding redundancy

**Fig 4:**
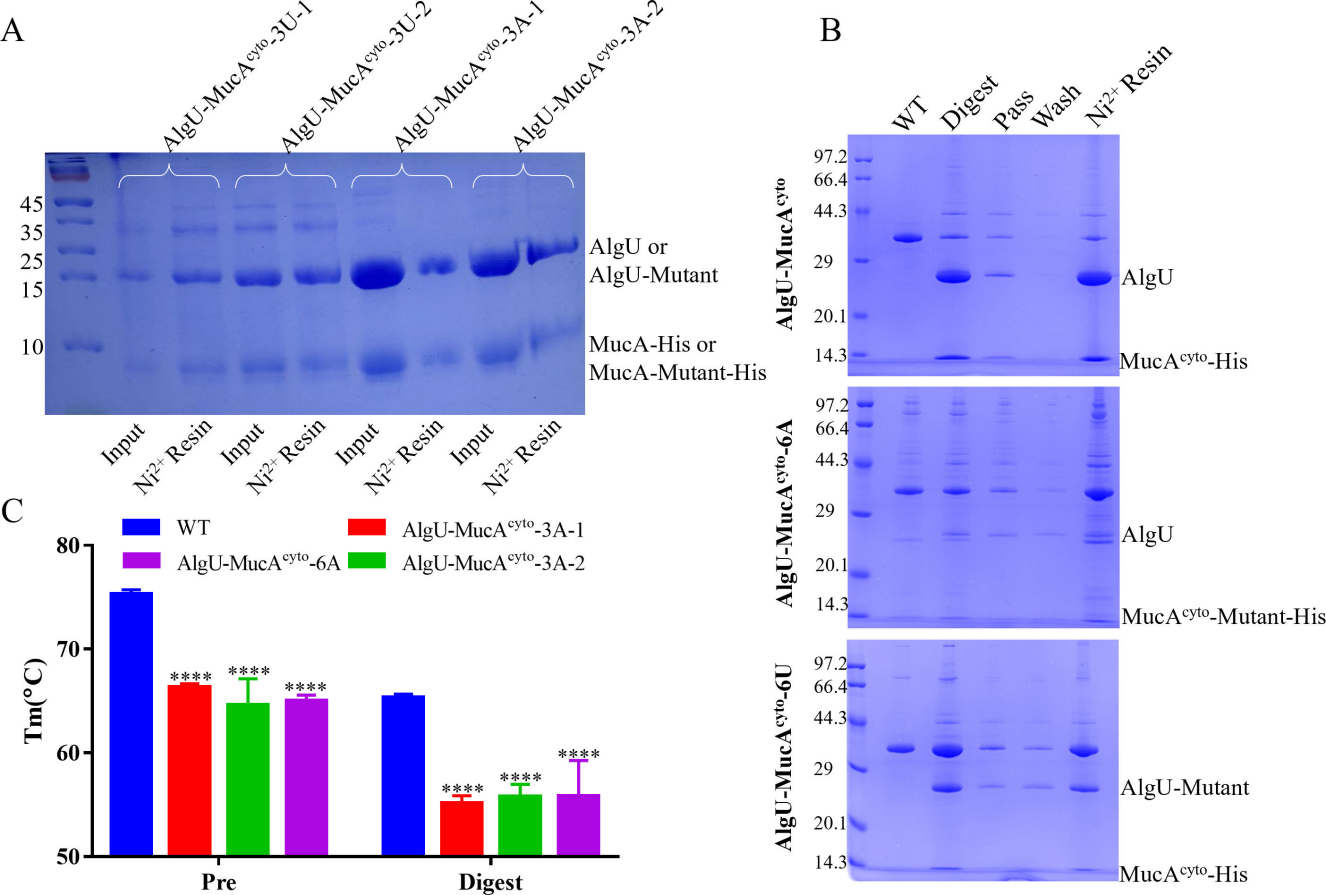
Identification of key residues crucial for AlgU-MucA^cyto^ complex binding and thermal stability. (**A and B**) Nickel affinity pull-down assays evaluating AlgU-MucA^cyto^ complex binding after HRV 3C protease cleavage. Following digestion with HRV 3C protease to remove affinity tags, protein complexes were incubated with nickel resin to pull-down components still containing the uncleaved His-tag. The binding efficiency of the cleaved partner was assessed by SDS-PAGE of the pull-down fractions. Panel A tests triple-cluster MucA^cyto^-targeted mutants AlgU-MucA^cyto^-3A-1 (E8A/E17A/E22A) and AlgU-MucAcyto-3A-2 (R37A/R42A/R47A), and AlgU-targeted mutants AlgU-MucA^cyto^-3U-1 (R150A/E151A/Y157A) and AlgU-MucA^cyto^-3U-2 (R172A/R177A/R179A). Panel B tests combinatorial six-site mutants: MucA^cyto^-targeted AlgU-MucAcyto-6A (E8A/E17A/E22A/R37A/R42A/R47A) and AlgU-targeted AlgU-MucA^cyto^-6U (R150A/E151A/Y157A/R172A/R177A/R179A). (**C**) Differential scanning fluorimetry (DSF) analysis of thermal stability (Tm). The melting temperature *T_m_* of the wild-type AlgU-MucA^cyto^ complex and MucA^cyto^-targeted mutants (AlgU-MucA^cyto^-3A-1, AlgU-MucA^cyto^-3A-2, and AlgU-MucA^cyto^-6A) was determined by DSF. Complexes were mixed with AGSYPRO Orange dye, and the fluorescence signal (excitation at 470 nm, emission at 570 nm) was monitored as the temperature increased. The inflection point of the resulting thermal denaturation curve defines the *T_m_*, where mutations causing decreased *T_m_* relative to wild type indicate destabilization of the complex. *, *P*<0.05; **, *P*<0.01; ***, *P*<0.001.

Based on prior analyses, the interaction between AlgU and MucA is primarily localized at two distinct binding interfaces, mediated predominantly by charged glutamic acid (E) and arginine (R) residues. To characterize key residues driving AlgU-MucA binding *in vitro*, we constructed expression plasmids encoding multiple mutations and expressed them in *E. coli* (Fig. S7). Four triple-site mutant variants of AlgU-H3H-MucA^cyto^ were generated: MucA^cyto^-targeted mutants AlgU-MucA^cyto^-3A-1 (E8A/E17A/E22A) and AlgU-MucA^cyto^-3A-2 (R37A/R42A/R47A), alongside AlgU-targeted mutants AlgU-MucA^cyto^-3U-1 (R150A/E151A/Y157A) and AlgU-MucA^cyto^-3U-2 (R172A/R177A/R179A) (Fig. S7). Subsequent HRV 3C proteolytic cleavage followed by pull-down assays revealed that His-tagged MucA^cyto^ could robustly capture non-tagged AlgU across all four triple-mutant variants (Fig. 4A), indicating intact complex formation. Expanding our approach, we constructed six-site combinatorial mutants for both AlgU and MucA^cyto^ (Fig. S7). Despite this extensive mutagenesis, pull-down experiments demonstrated persistent AlgU and MucA^cyto^ binding for both AlgU-MucA^cyto^-6A and AlgU-MucA^cyto^-6U mutants (Fig. 4B), providing compelling evidence that their high-affinity interaction resists disruption even by large-scale, localized residue substitutions.

To further evaluate the functional significance of these residues, we assessed mutant thermal stability using DSF. While mutants for AlgU universally failed to yield quantifiable signals, all viable MucA^cyto^ mutants exhibited significantly reduced melting temperatures (*T*_*m*_) relative to wild type in both fusion-protein and post-digestion states (Fig. 4B; Fig. S8). These findings indicate that although the mutations do not abrogate binding, they substantially destabilize the AlgU-MucA^cyto^ complex. This thermal destabilization indirectly confirms the critical role of these residues in maintaining the structural integrity of the complex. These data collectively establish that localized mutations at key AlgU-MucA^cyto^ interfacial residues do not disrupt complex formation but are essential for maintaining structural integrity, as evidenced by significant thermal destabilization.

### SspB acts as a potential adaptor for AlgU-MucA^cyto^ recognition and ClpX/ClpP degradation

**Fig 5:**
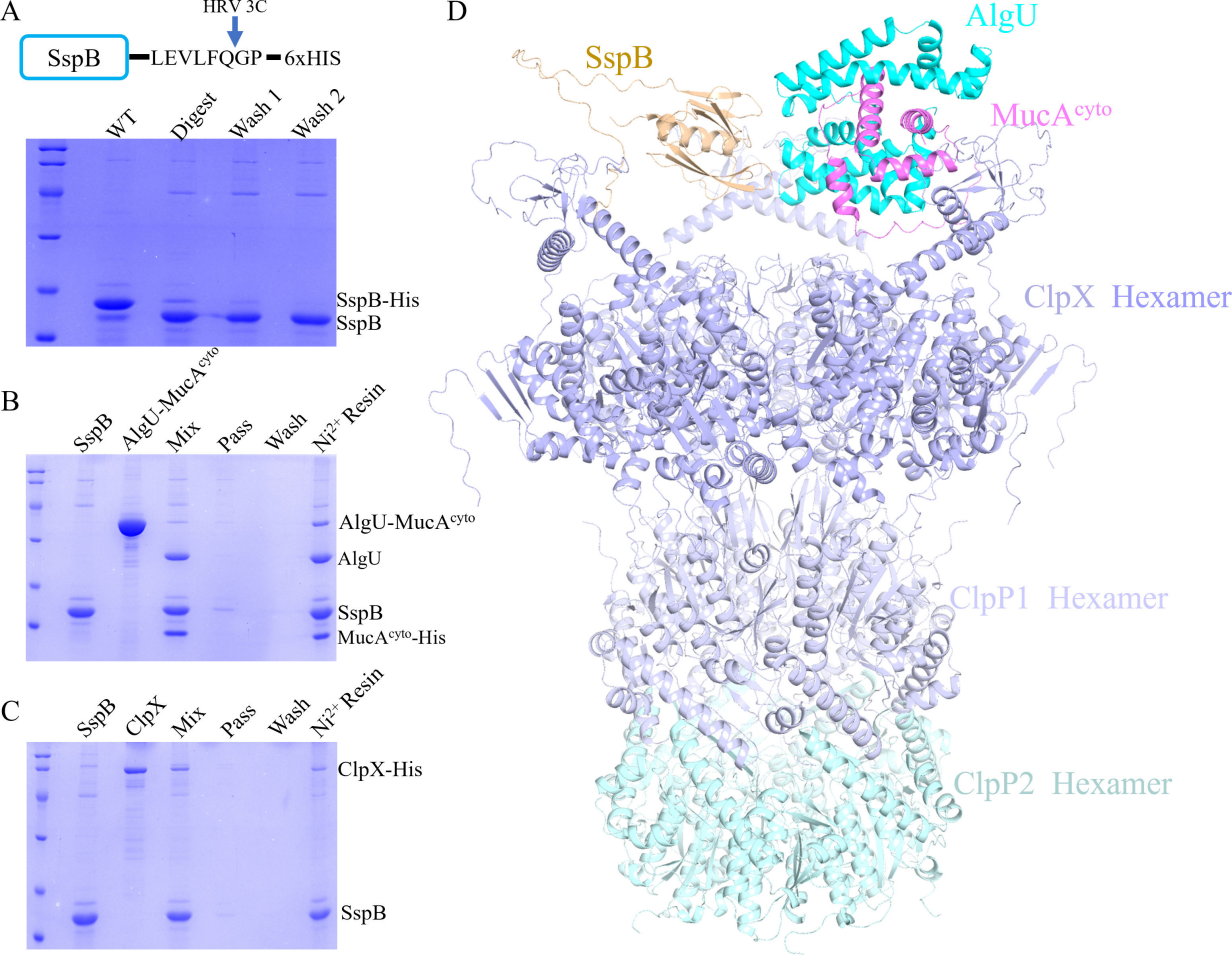
Validation of SspB interactions with the AlgU-MucA^cyto^ complex and ClpX. (**A**) SDS-PAGE analysis of His-tag removal from SspB. Purified SspB-His protein before and after digestion by HRV 3C protease. Successful tag excision is confirmed by reduced molecular weight. (**B**) Nickel pull-down assay demonstrating SspB binding to the AlgU-MucA^cyto^ complex. Untagged SspB co-purifies with nickel resin when incubated with His₆-tagged AlgU-H₃H-MucA^cyto^. SspB retention indicates stable interaction despite partial AlgU-H₃H-MucA^cyto^ complex cleavage by residual HRV 3C protease. (**C**) Nickel pull-down assay confirming SspB-ClpX interaction. Untagged SspB is efficiently pulled down by His₆-tagged ClpX. (**D**) Structural model of the SspB/AlgU-MucA^cyto^/ClpX/ClpP degradation complex predicted by AlphaFold3.

Recognition of the AlgU-MucA^cyto^ complex by adaptor proteins constitutes a critical step in AlgU activation (18). These adaptors modulate interactions between specific substrates and AAA+ proteases like ClpX/ClpP, thereby controlling substrate recognition and degradation hierarchy (19). Studies demonstrate that starvation protein B (SspB) delivers substrates to the AAA+ protease ClpX/ClpP (20–22), with SspB functionally defined as a positive regulator of proteolysis and exhibiting tight binding to ClpX (Fig. S9). To validate whether SspB acts as a key adaptor delivering AlgU-MucA^cyto^ to ClpX/ClpP for MucA^cyto^ degradation, we constructed SspB-His (Fig. S9) and removed the His-tag via HRV 3C protease cleavage for subsequent pull-down assays examining its binding to AlgU-MucA^cyto^ and ClpX (Fig. 5A).

Our results reveal that untagged SspB co-purified with nickel resin when incubated with AlgU-H3H-MucA^cyto^. Despite partial cleavage of AlgU-H3H-MucA^cyto^ by residual HRV 3C protease, SspB was robustly pulled down, likely mediated by dual interactions with AlgU-H3H-MucA^cyto^ and MucA^cyto^-His (Fig. 5B). Concurrently, untagged SspB also bound ClpX-His in parallel pull-down assays (Fig. 5C).

We subsequently employed AlphaFold3 to simulate the recognition complex comprising SspB, AlgU-MucA^cyto^, and the ClpX/ClpP protease. Structural analysis revealed that the N-terminal domain of SspB consists of one α-helix and six β-strands, with its apical region engaging the hairpin motif of AlgU (Fig. 5D). Notably, residues 99–135 at the C-terminus of SspB form a disordered region whose terminal peptide segment mediates ClpX binding. Although the C-terminal extension of MucA^cyto^ does not protrude into the central pore of the hexameric ClpX ring, this simulated structure provides mechanistic insights into how SspB recognizes AlgU-MucA^cyto^ and delivers it to ClpX/ClpP for degradation. These data suggest that proteolysis of MucA^cyto^ likely initiates at its C-terminal extended region, ultimately leading to AlgU release. These findings collectively identify SspB as the functional adaptor that directs AlgU-MucA^cyto^ to ClpX/ClpP for the targeted degradation of MucA^cyto^.

### Structure-guided mutagenesis and BLI validation support a dual-interface adaptor role of SspB

**Fig 6:**
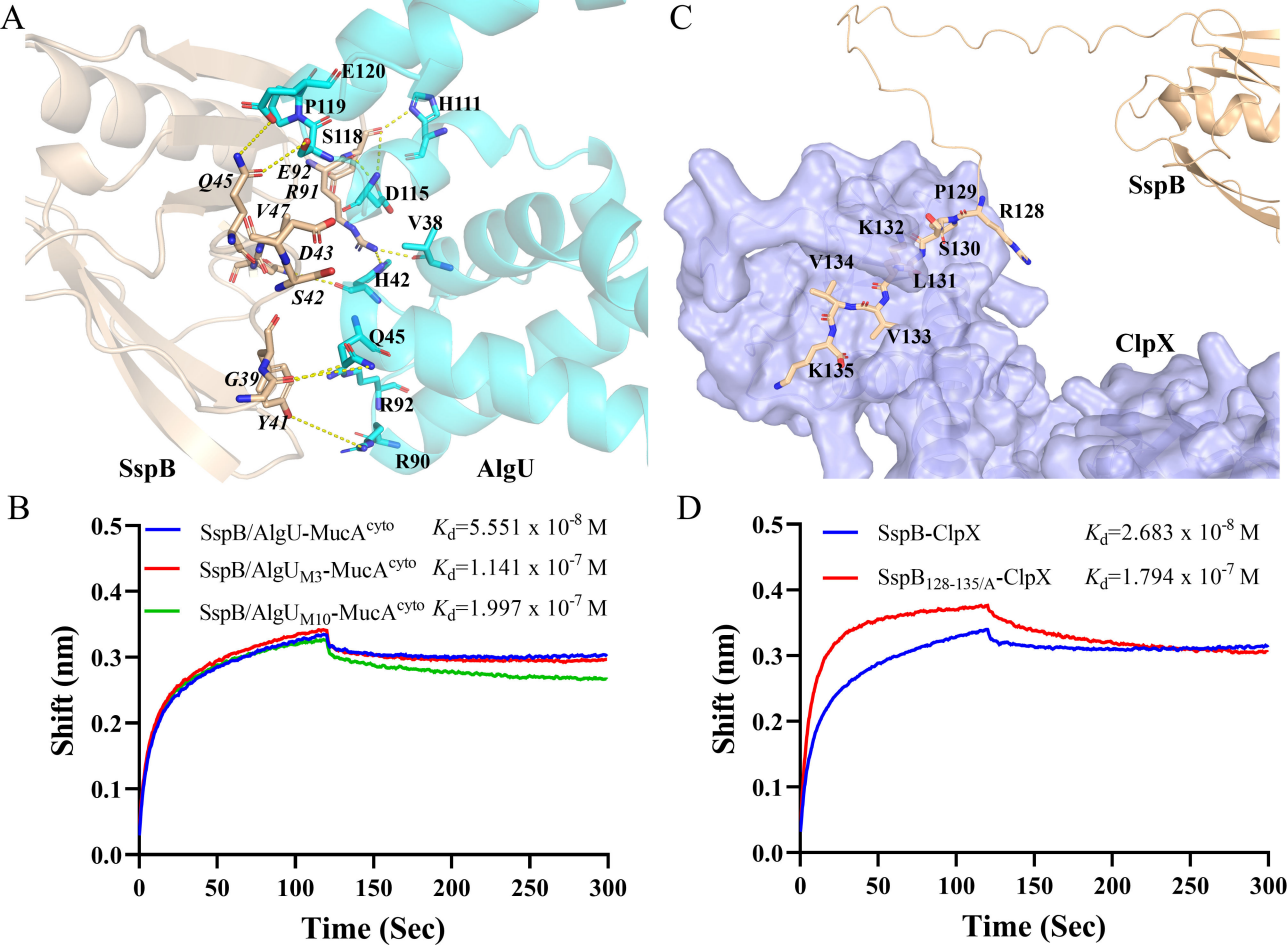
Structure-guided validation of SspB interactions with the AlgU-MucA^cyto^ complex and ClpX by BLI. (**A**) Close-up view of the predicted SspB-AlgU interface. Residues on AlgU implicated in SspB recognition are shown as sticks, including V38, H42, Q45, R90, R92, H111, D115, and S118/P119/E120. Dashed lines indicate predicted polar interactions at the interface. (**B**) Bio-layer Interferometry (BLI) determined the affinity of SspB binding to the AlgU-MucA^cyto^ complex. Wild-type AlgU-MucA^cyto^ is compared with two AlgU interface mutants: AlgU_M3_-MucA^cyto^ (V38A/H111A/D115A) and AlgU_M10_-MucA^cyto^ (V38A/H111A/D115A/H42A/Q45A/R90A/R92A/S118A/P119A/E120A). The corresponding *K*_*d*_ values are indicated. (**C**) Structural model of SspB docking to ClpX, highlighting the SspB C-terminal tail (residues 128–135) contacting the ClpX surface. (**D**) BLI determined the affinity of SspB binding to ClpX. Wild-type SspB is compared with the alanine-substitution mutant SspB_128-135A_ (residues R128–K135 replaced by alanine). The corresponding *K*_*d*_ values are indicated.

To better define the adaptor function of SspB and to validate the reliability of the complex model, we first analyzed the predicted direct interaction interface between SspB and the AlgU-MucA^cyto^ complex and designed interface-perturbation mutants accordingly. The structural model indicates that R91/E92 of SspB occupy the core of the AlgU-contact region and form an interaction network dominated by hydrogen bonding and electrostatic contacts with AlgU residues V38, H111, and D115. In addition, AlgU residues H42, Q45, R90, R92, and S118/P119/E120 lie in close proximity to SspB at the interface and are predicted to contribute tight van der Waals packing together with local polar contacts (Fig. 6A), collectively defining the key surface for SspB recognition of AlgU. Guided by this interface, we quantified binding of wild-type SspB to the preassembled AlgU-MucA^cyto^ complex by BLI, yielding a *K*_*d*_ of 5.551 × 10^−^⁸ M (Fig. 6B). We then generated two structure-guided AlgU mutants within the AlgU-MucA^cyto^ complex to test interface contributions. The first mutant, AlgU_M3_-MucA^cyto^, carries V38A/H111A/D115A, designed to perturb the key polar/electrostatic node in the model. The second, a stronger interface-perturbation mutant AlgU_M10_-MucA^cyto^, extends the M3 substitutions by additionally replacing H42, Q45, R90, R92, S118, P119, and E120 with alanine, targeting residues predicted to contribute primarily through van der Waals packing with local polar contributions. Compared with wild-type AlgU-MucA^cyto^, both mutants reproducibly weakened SspB binding, with *K*_*d*_ values shifting to 1.141 × 10^−^⁷ M and 1.997 × 10^−^⁷ M for AlgU_M3_-MucA^cyto^ and AlgU_M10_-MucA^cyto^, respectively (Fig. 6B). Together, these data provide quantitative support for the model-predicted AlgU-MucA^cyto^ recognition surface and anchor the substrate-recognition function of SspB to the AlgU-facing interface within the AlgU-MucA^cyto^ complex.

After establishing that SspB recognizes the AlgU-MucA^cyto^ complex via the AlgU interface, we next tested the model prediction that SspB engages ClpX through its C-terminal disordered tail. The model suggests that the SspB C-terminal segment (R128–K135) extends toward a protruding surface of dimeric ClpX and forms an extended contact strip with the ClpX surface (Fig. 6C). This architecture is consistent with an adaptor mechanism in which one end uses a folded domain to recognize the substrate complex, whereas the other end uses a flexible/disordered tail to dock onto the protease machinery. Accordingly, BLI measurements showed that wild-type SspB binds ClpX with a *K*_*d*_ of 2.683 × 10^−^⁸ M (Fig. 6D). To directly assess the contribution of the tail residues (R128–K135), we generated SspB_128–135A_, in which residues R128–K135 were replaced by alanine. This mutant markedly weakened binding to ClpX, with the *K*_*d*_ shifting to 1.794 × 10^−^⁷ M (Fig. 6D), indicating that ClpX engagement depends primarily on the SspB C-terminal disordered region. Thus, the structural framework and BLI quantification converge on two independent interfaces, providing structure–quantitation concordant evidence for a dual-interface adaptor role of SspB: it recognizes the AlgU-MucA^cyto^ complex while docking ClpX/ClpP via its C-terminal tail, thereby enabling substrate delivery and targeted degradation.

## DISCUSSION

Cystic fibrosis (CF) lung disease, a common clinical disease caused by *P. aeruginosa* infection, is primarily characterized by the accumulation of a large amount of mucoid secretions in the patient’s bronchial tract, leading to tracheal blockage (23, 24). Most of this mucoid secretion is an extracellular polysaccharide secreted by *P. aeruginosa*. In this study, we constructed a stress-responsive function σ^ECF^ factor AlgU knockout strain and observed a slight reduction in pyocyanin and a significant reduction in alginate (25–27). Notably, the modest change in pyocyanin is likely indirect. Previous work has linked AlgR to pyocyanin control through regulatory connections involving CzcR, and AlgU-dependent modulation of AlgR could contribute to the observed trend (28). In contrast, the marked decrease in alginate production is consistent with AlgU contributing to alginate-associated mucoid phenotypes relevant to *P. aeruginosa* infection in CF lung disease (29). Importantly, the complementation results should be interpreted cautiously. In this study, *algU* was complemented in *trans* using a multicopy plasmid driven by the IPTG-inducible P_Tac_ promoter, which can elevate AlgU levels relative to physiological single-copy expression. Therefore, phenotypic shifts observed upon complementation, especially when exceeding the wild-type baseline, are best considered a supportive context for AlgU involvement rather than a strict restoration of native regulation. To more stringently assess AlgU-dependent phenotypes under physiological dosage, single-copy chromosomal complementation (or other single-copy systems) would be preferable and will be pursued in future work. In addition, deletion of AlgU increased cell invasiveness, which may reflect altered engagement of host-cell interaction pathways. In line with this possibility, *algU* inactivation has been reported to increase systemic virulence in acute mouse infection models (15). Rather than directly attributing this phenotype to reduced alginate production, we speculate that loss of AlgU may shift the regulatory balance toward an “acute-infection” program that favors host-cell interactions, potentially through de-repression of invasion-associated determinants such as the type III secretion system (T3SS) and related acute virulence pathways (30, 31). This interpretation is consistent with the reciprocal regulation of acute versus chronic persistence traits in *P. aeruginosa* and with prior observations that *algU* inactivation can enhance virulence in acute infection settings (5).

Although the crystal structure of the AlgU-MucA^cyto^ complex has been determined (17), AlgU and MucA are challenging to express and purify when produced individually due to their low expression levels and poor purification quality. In this study, we overcame these obstacles by fusing AlgU to MucA via an H3H-tag, which allowed high-yield, stable expression of the fusion protein. Subsequently, we employed HRV3C protease to cleave and obtain the AlgU-MucA complex. This integrated approach establishes a robust strategy for preparing and structurally characterizing recalcitrant protein complexes that resist conventional expression methods.

Through structural comparison and overall structural analysis of the AlgU-MucA^cyto^ complex with the RopE-RseA^cyto^ (PDB code: 1OR7) complex in *E. coli*, we found a consistent binding mode, indicating functional conservation between *P. aeruginosa* and *E. coli*. Furthermore, in the structure of the AlgU-MucA^cyto^ complex, we identified key amino acid sites on MucA, E4, E8, E17, E22, and R37, R42, R47, which form an extensive and tight hydrogen bond and charge–charge interaction network with Q9, R177, D150, E151, Y157, R172, R177, R179, D183, etc., in AlgU. It is noteworthy that in the structure, we found that the sites E4, E8, and E17 on α1 and E22 on α2 of MucA, through molecular interaction, firmly lock the opening position of the hairpin-like structure of AlgU-N and AlgU-C, further stabilizing the binding of AlgU with MucA^cyto^. Notably, this interaction is distributed across a broad, highly redundant electrostatic network. Accordingly, clustered alanine substitutions at selected interfacial nodes (e.g., the triple- and sextuple-site variants) can leave sufficient residual contacts to preserve complex formation in pull-down assays, yet markedly weaken the cooperative hydrogen-bonding/salt-bridge scaffold that rigidifies the bound state. This “binding-stability uncoupling” provides a plausible explanation for why the mutants retain association but exhibit substantially reduced thermal stability in DSF. Importantly, we acknowledge that the triple and sextuple alanine variants were designed as systematic interface-perturbation tools to probe the robustness of the interaction network and are not intended to represent naturally occurring combinations; assessment of *in vivo* functional consequences will require chromosome-level mutagenesis/complementation and phenotypic assays in future work.

It is noteworthy that, despite the identification of key amino acid residues from the structure, pull-down assays confirmed that neither triple nor even combinatorial sextuple mutations within AlgU or MucA^cyto^ disrupted complex formation between AlgU and MucA^cyto^. This persistent binding, validated even after HRV 3C cleavage, demonstrates a robust interaction network. However, DSF revealed a critical distinction: while binding remained intact, these mutations significantly destabilized the complex. Mutant complexes exhibited substantially decreased melting temperatures (*T*_*m*_) in both fusion-protein and post-digestion states. This thermal destabilization shows these residues are essential for maintaining the structural rigidity of the bound complex, even though they are redundant for complex formation itself. This dual characteristic, resilient binding coupled with strict stability requirements, ensures that AlgU is not inadvertently activated due to point mutations in MucA. It powerfully underscores the essential, non-redundant role of the ClpX/ClpP protease complex-mediated degradation of MucA^cyto^ in the controlled activation of AlgU within the MucA-RIP signaling pathway (16, 32, 33). Given previous reports that uncontrolled AlgU overactivation (due to MucA mutation or deletion) causes growth inhibition and death in *P. aeruginosa*, we propose that this extensive interactive network and its specific stability requirements reflect a key evolutionary adaptation. The resilience to mutation safeguards against accidental activation, while reliance on regulated proteolysis ensures precise control under stress, highlighting the fundamental importance of the MucA-RIP pathway for *P. aeruginosa* survival.

At the systems level, AlgU output is also subject to additional regulatory inputs beyond the proteolytic checkpoint described above. Previous work in *P. aeruginosa* showed that SspA (together with other anti-σ^70^ factors) can indirectly enhance AlgU-driven mucoidy by attenuating RpoD (σ^70^) activity and thereby reshaping sigma-factor competition (34). Moreover, in *Pseudoalteromonas sp. R3*, SspA was reported to upregulate *algU* expression and promote exopolysaccharide production and biofilm formation (35), supporting a predominant role of SspA at the transcriptional/regulatory tier. In contrast, SspB is functionally annotated in Pseudomonas genomes as a ClpXP protease specificity-enhancing factor, suggesting a post-translational adaptor role. Consistent with this functional partitioning, our structural framework and BLI measurements support that SspB recognizes the preassembled AlgU-MucA^cyto^ complex and uses its C-terminal disordered region to engage ClpX/ClpP, thereby facilitating targeted degradation of MucA^cyto^. Taken together, SspA and SspB may act at complementary layers—sigma competition/AlgU expression control versus protease recruitment and substrate delivery, to coordinate AlgU pathway activation across environmental transitions.

Furthermore, we identified SspB as a key adaptor protein facilitating the targeted degradation of the AlgU-MucA^cyto^ complex by the ClpX/ClpP protease. Pull-down assays demonstrated the ability of SspB to interact with both AlgU-MucA^cyto^ and ClpX. AlphaFold3 modeling provided a structural framework for this recognition, suggesting SspB engages AlgU via its N-terminal domain while utilizing a disordered C-terminal region to dock onto ClpX. This positions SspB as a crucial bridge, delivering the substrate complex to the protease. The model also implies that degradation initiation likely occurs at the exposed C-terminal extension of MucA^cyto^, ultimately leading to AlgU release—a key step in the activation pathway. The identification of SspB as the functional adaptor completes a critical link in the regulatory pathway controlling AlgU activity. To further establish the biological relevance of this adaptor model, future studies will combine Δ*sspB*-based phenotypic analyses with *in vitro* reconstituted ClpXP degradation assays to directly assess SspB-dependent substrate delivery and proteolysis under physiologically relevant stress conditions.

In conclusion, our data reveal a sophisticated mechanism governing the AlgU-MucA^cyto^ complex. Binding redundancy at the primary interfaces ensures complex formation persists even under significant residue perturbation, while specific critical residues are indispensable for conferring structural stability. This stable complex is then recognized by the adaptor SspB, which orchestrates its delivery to ClpX/ClpP for the degradation of MucA^cyto^, thereby activating AlgU. This work significantly advances our understanding of the molecular details underlying this critical bacterial stress response pathway.

## MATERIALS AND METHODS

### Gene disruption of *algU* in *P. aeruginosa*

A *sacB*-based two-step allelic exchange strategy was employed to construct full-length *algU* and knockout of *P. aeruginosa* (36). The upstream and downstream (400 bp) PCR fragments of *algU* were ligated by overlap extension PCR with the gene-specific primers. Then, target fragments were recombined into the linearized DNA fragment of pEX18Gm with the Ligation-Free cloning system (5× Ligation-Free cloning master Mix, abm). This vector was transformed into *E. coli* S17-1-competent cells and then mobilized into *P. aeruginosa* strains PAO1/PA14, respectively. Following the principle of two-step allelic exchange, colonies were screened for nutritional selection on pseudomonas isolation agar (PIA) + 30 mg/mL gentamicin resistance and a counter-selection on no-salt LB (NSLB) + 10% (wt/vol) sucrose, with gentamicin resistance (50 mg/mL), which typically indicated a double crossover event and thus the occurrence of gene replacement (37). The Δ*algU* strains were further confirmed by PCR and DNA sequencing.

### The Δ*algU* complementation plasmid construction

To construct the complementation plasmid pME6032-algU, DNA fragments of *algU* were amplified by polymerase chain reaction (PCR) from the *P. aeruginosa* genome and were cloned into the XbaI and Hind-III sites of plasmid pME6032 (38) using the Blunting Kination Ligation Kit (TaKaRa). After sequencing, the complementation plasmids were transformed into *E. coli* S17-1-competent cells and then mobilized into *P. aeruginosa* PAO1/PA14-Δ*algU* strains by conjugation. All strains were then screened by on PIA supplemented with 150 μg/mL tetracycline, PCR, and DNA sequencing. All complementation strains were induced by the addition of 0.5 mM IPTG.

### Alginate assay

GDP-mannuronic acid is an early step in the biosynthesis of alginate (9). Therefore, the alginate quantification assay was performed using the uronic acid concentrations, which were determined at OD_520_. *P. aeruginosa* cultures were grown on PIA plates for 36 hours. Cells were harvested and measured at OD_600_. Cell pellets were removed by centrifugation. An amount of 15 μg/mL of DNase and RNase was added to the supernatant, and this mixture was incubated at 37°C for 6 hours. After nuclease digestion, Protease K was added to a final concentration of 20 μg/mL, and the solution was incubated again for 18 hours at 37°C in a shaking incubator.

For further purification, the supernatants containing dissolved alginate were (dialysis membranes) dialyzed in 10 mM Tris-HCl (pH 7.6) buffer at 4°C overnight. An amount of 100 µL of the dialysis fraction was mixed with 1.0 mL of borate-sulfuric acid reagent (100 mM H_3_BO_3_ in concentrated H_2_SO_4_) and 100 µL of carbazole reagent (0.1% in ethanol). The mixture was heated to 55°C for 30 minutes, and the OD_520_ was determined (39).

An alginate standard curve was built using sodium alginate and was determined as micrograms of uronic acid per milligram of cell weight (wet weight) or the relative amounts of alginate (%) (9, 40, 41).

### Pyocyanin and pyoverdine assay

Pyocyanin was extracted from culture supernatants and measured using previously reported methods (28, 42). Briefly, 3 mL chloroform was added to 10 mL cell culture supernatant. After extraction, the chloroform layer was transferred to a fresh tube and mixed with 1 mL of 0.2 M HCI. After centrifugation, the top layer was removed, and its OD_520_ was measured. Pyocyanin concentrations were determined by multiplying the OD_520_ by 17.072.

Pyoverdine production by *P. aeruginosa* growing in iron-limited succinate medium was assayed by measuring the absorbance of culture supernatants at 400 nm using UV-visible spectrophotometry, as previously described (43). A single colony of WT or mutant bacteria was inoculated into 3 mL of LB media and grown overnight for 24 hours at 37°C under agitation (220 rpm). The next day, dilute 1 mL of this culture in 10 mL of succinate medium, and run a 24-hour culture at 30°C under agitation (220 rpm). The following day, the culture was diluted in succinate (1 mL in 10 mL medium) again and allowed them to grow for 24 hours at 30°C under agitation. Cells were pelleted by centrifugation at 12,000 rpm for 3 minutes, and the supernatant was diluted 10-fold in succinate medium. Pyoverdine content was determined by measurement of absorption at 400 nm, and the pyoverdine production (normalized to cell number as measured by A600) of WT or mutant was calculated.

### Plasmid construction, protein expression, and purification

The AlgU (full length) and MucA^cyto^ (1–80) were fused together by using connectors containing HRV3C restriction (LEVLFQ↓GP) and His_6_-Tag (marked as H3H-Tag), and the plasmid was named pET21-AlgU-H3H-MucA^cyto^. Then, pET21-AlgU-H3H-MucA^cyto^ was transfected into BL21 (DE3)-competent cells, and the protein expression was induced by 0.5 mM isopropyl-D-thiogalactoside (IPTG) at 18℃ and 220 rpm. The proteins were purified in general through two steps: Ni-NTA chromatography (GE Healthcare, USA) and gel filtration chromatography (GFC). AlgU-H3H-MucA^cyto^ protein was digested by HRV3C protease to destroy the H3H-tag, and then the residual HRV3C protease was removed by GFC column, and a high-purity AlgU-MucA^cyto^ complex was obtained.

### Proteolytic cleavage of AlgU-H3H-MucA^cyto^ fusion protein

For proteolytic cleavage, the AlgU-H3H-MucA^cyto^ fusion protein containing an HRV 3C recognition site was mixed with HRV 3C protease at a mass ratio of 100:1 in cleavage buffer (50 mM Tris-HCl, 150 mM NaCl, pH 7.5). The reaction mixture was incubated at 4°C for 16 hours. Cleavage efficiency was assessed by SDS-PAGE and Ni²^+^-NTA affinity chromatography.

### Ni²^+^-NTA affinity chromatography for cleavage product separation

Following the cleavage reaction, the mixture was loaded onto a Ni²^+^-NTA affinity column pre-equilibrated with buffer containing 25 mM Tris-HCl, 150 mM NaCl, and 10 mM imidazole (pH 7.5). The column was washed with three column volumes of equilibration buffer to remove non-specifically bound proteins. The flow-through, wash fractions, and resin-bound material were collected and analyzed by 12% SDS-PAGE to verify the cleavage efficiency and separation of cleaved products from uncleaved fusion protein and HRV 3C protease.

### Thermal stability analysis by DSF

The thermal stability of proteins was assessed by DSF using a QuantStudio 5 real-time PCR system (Applied Biosystems). Reaction mixtures (20 μL) contained 2 μg of protein sample (either intact AlgU-MucA^cyto^ or the cleavage mixture) and AGSYPRO Orange dye (Accurate Biotechnology [Hunan] Co., Ltd) at a final concentration of 1×. A control sample containing 0.02 μg of HRV 3C protease alone was also included. Each condition was prepared in quadruplicate in a 96-well PCR plate. Fluorescence was recorded from 25°C to 99°C with a temperature ramp rate of 1°C/min (excitation at 470 nm, emission at 570 nm). Melting temperatures (*T_m_*) were determined as the inflection point of the fluorescence derivative curve (−d*F*/d*T*).

### Pull down

Interaction of SspB with AlgU-MucA and ClpX. The C-terminal 6× His tag of purified SspB-His was removed by HRV 3C protease digestion, and tag-free SspB was further purified by Ni-NTA chromatography. For binding assays, tag-free SspB was individually mixed with His-tagged target proteins at a molar ratio of 5:1, including the AlgU-MucA^cyto^-6His complex and monomeric ClpX-6His. The mixtures were incubated in binding buffer (25 mM Tris-HCl, pH 7.0, 150 mM NaCl) at 4°C for 2 hours and then loaded onto a Ni-NTA column pre-equilibrated with the same buffer supplemented with 20 mM imidazole. Flow-through and wash fractions were collected, and a small aliquot of the resin was retained as the resin-bound fraction. All samples were analyzed by SDS-PAGE to assess the specific binding of SspB to the target proteins.

Interaction of SspB with AlgU-MucA^cyto^ mutant complexes. Tag-free SspB was incubated separately with three AlgU-MucA^cyto^-6His complexes: (i) wild-type AlgU-MucA^cyto^-6His; (ii) AlgU-MucA^cyto^-3U/6U-6His (AlgU mutants); and (iii) AlgU-MucA^cyto^-3A/6A-6His (MucA mutants). Incubation and purification conditions were identical to those described above. Binding outcomes were compared by SDS-PAGE analysis.

### BLI measurements

BLI experiments were performed using a GatorPrime instrument from GatorBio. All assays were run at 30°C with continuous 1,000 rpm shaking. The assay buffer consisted of phosphate-buffered saline (PBS), 0.02% Tween-20, adjusted to pH 7.5. Biotinylated MMLV RT-SV proteins were tethered on streptavidin (SA-XT) biosensors (GatorBio) by dipping sensors into protein solutions. SA-XT biosensors were then washed in assay buffer for 120 seconds to eliminate nonspecifically bound protein and establish stable baselines. Then, the SA-XT biosensor was dipped into various concentrations of AlgU-MucA^cyto^ or ClpX for 120 seconds to record the association phase. Then, the sensors were placed into wells containing measuring buffer and tag-free SspB for 180 seconds to record the dissociation phase. All the data were analyzed by Gator Bio data analysis software. The equilibrium dissociation constant (*K*_*d*_) values were calculated.

### Crystallization, data collection, and structural determination

Crystallization screens were carried out by mixing AlgU-MucA^cyto^ protein complex with reservoir buffer at 18°C using the hanging-drop vapor diffusion method. Crystals were obtained in the solution of PEG F6: 10% 2-propanol, 0.1 M BICINE pH 8.5 NaOH, 30% PEG1500.

Diffraction data were collected on beamline BL18U/BL19U of the Shanghai Synchrotron Radiation Facility (SSRF), China. Data were integrated, scaled, and merged with the HKL2000 program package. The structure of AlgU-MucA^cyto^ was determined and refined by using PHENIX. The final refinement statistics for these structures are summarized in Fig. 2B.

### A549 cell invasion assays

Biofilm-specific antibiotic resistance assays referred to a previous method (44). A549 cells (obtained from ATCC) were grown in RPMI 1640 medium containing 10% (vol/vol) fetal bovine serum (FBS; Gibco, Auckland, New Zealand) and 1% antibiotics (penicillin and streptomycin) in 5% CO_2_ at 37°C. After seeding the suspension cultures into plates, cells were washed with PBS (pH 7.2) and changed to antibiotic-free medium immediately before infection. The cells were washed with PBS and incubated for 1 hour in RPMI 1640 medium containing 150 μg/mL of gentamicin. The monolayer cells were then washed with PBS and lysed with 0.5% Triton X-100 for 10–20 minutes, and appropriate dilutions were plated on PIA plates to determine the number of viable intracellular bacteria.

## Data Availability

All data relevant to this study are supplied in the article and supplemental file or are available from the corresponding author upon request. The atomic coordinates and structure factors for the AlgU-MucA^cyto^ complex have been deposited in the Protein Data Bank (PDB, https://www.rcsb.org/) and are publicly available under accession code 8Z6G.

## SUPPLEMENTAL MATERIAL

Supplemental Figures mbio.00148-26-s0001.docx Fig. S1 to S9.

ASM does not own the copyrights to Supplemental Material that may be linked to, or accessed through, an article. The authors have granted ASM a non-exclusive, world-wide license to publish the Supplemental Material files. Please contact the corresponding author directly for reuse.

## References

[1] Stover CK, Pham XQ, Erwin AL, Mizoguchi SD, Warrener P, Hickey MJ, Brinkman FSL, Hufnagle WO, Kowalik DJ, Lagrou M, et al.. 2000. Complete genome sequence of Pseudomonas aeruginosa PAO1, an opportunistic pathogen. Nature 406:959–964. doi:10.1038/35023079 10984043

[2] Silby MW, Winstanley C, Godfrey SAC, Levy SB, Jackson RW. 2011. Pseudomonas genomes: diverse and adaptable. FEMS Microbiol Rev 35:652–680. doi:10.1111/j.1574-6976.2011.00269.x 21361996

[3] Kwan T, Liu J, Dubow M, Gros P, Pelletier J. 2006. Comparative genomic analysis of 18 Pseudomonas aeruginosa bacteriophages. J Bacteriol 188:1184–1187. doi:10.1128/JB.188.3.1184-1187.2006 16428425 PMC1347338

[4] Arora SK, Neely AN, Blair B, Lory S, Ramphal R. 2005. Role of motility and flagellin glycosylation in the pathogenesis of Pseudomonas aeruginosa burn wound infections. Infect Immun 73:4395–4398. doi:10.1128/IAI.73.7.4395-4398.2005 15972536 PMC1168557

[5] Huang H, Shao X, Xie Y, Wang T, Zhang Y, Wang X, Deng X. 2019. An integrated genomic regulatory network of virulence-related transcriptional factors in Pseudomonas aeruginosa. Nat Commun 10:2931. doi:10.1038/s41467-019-10778-w 31270321 PMC6610081

[6] Ippolito M, Misseri G, Catalisano G, Marino C, Ingoglia G, Alessi M, Consiglio E, Gregoretti C, Giarratano A, Cortegiani A. 2021. Ventilator-associated pneumonia in patients with COVID-19: a systematic review and meta-analysis. Antibiotics (Basel) 10:545. doi:10.3390/antibiotics10050545 34067186 PMC8150614

[7] Yu H, Schurr MJ, Deretic V. 1995. Functional equivalence of Escherichia coli sigma E and Pseudomonas aeruginosa AlgU: E. coli rpoE restores mucoidy and reduces sensitivity to reactive oxygen intermediates in algU mutants of P. aeruginosa. J Bacteriol 177:3259–3268. doi:10.1128/jb.177.11.3259-3268.1995 7768826 PMC177019

[8] Maleki S, Mærk M, Hrudikova R, Valla S, Ertesvåg H. 2017. New insights into Pseudomonas fluorescens alginate biosynthesis relevant for the establishment of an efficient production process for microbial alginates. N Biotechnol 37:2–8. doi:10.1016/j.nbt.2016.08.005 27593394

[9] Li T, He L, Li C, Kang M, Song Y, Zhu Y, Shen Y, Zhao N, Zhao C, Yang J, Huang Q, Mou X, Tong A, Yang J, Wang Z, Ji C, Li H, Tang H, Bao R. 2020. Molecular basis of the lipid-induced MucA-MucB dissociation in Pseudomonas aeruginosa. Commun Biol 3:418. doi:10.1038/s42003-020-01147-1 32747658 PMC7400510

[10] Li T, Song Y, Luo L, Zhao N, He L, Kang M, Li C, Zhu Y, Shen Y, Zhao C, Yang J, Huang Q, Mou X, Zong Z, Yang J, Tang H, He Y, Bao R. 2021. Molecular basis of the versatile regulatory mechanism of HtrA-type protease AlgW from Pseudomonas aeruginosa. mBio 12:e03299-20. doi:10.1128/mBio.03299-20 33622718 PMC8545111

[11] Panmanee W, Su S, Schurr MJ, Lau GW, Zhu X, Ren Z, McDaniel CT, Lu LJ, Ohman DE, Muruve DA, Panos RJ, Yu HD, Thompson TB, Tseng BS, Hassett DJ. 2019. The anti-sigma factor MucA of Pseudomonas aeruginosa: dramatic differences of a mucA22 vs. a ΔmucA mutant in anaerobic acidified nitrite sensitivity of planktonic and biofilm bacteria in vitro and during chronic murine lung infection. PLoS One 14:e0216401. doi:10.1371/journal.pone.0216401 31158231 PMC6546240

[12] Intile PJ, Diaz MR, Urbanowski ML, Wolfgang MC, Yahr TL. 2014. The AlgZR two-component system recalibrates the RsmAYZ posttranscriptional regulatory system to inhibit expression of the Pseudomonas aeruginosa type III secretion system. J Bacteriol 196:357–366. doi:10.1128/JB.01199-13 24187093 PMC3911257

[13] Anthony M, Rose B, Pegler MB, Elkins M, Service H, Thamotharampillai K, Watson J, Robinson M, Bye P, Merlino J, Harbour C. 2002. Genetic analysis of Pseudomonas aeruginosa isolates from the sputa of Australian adult cystic fibrosis patients. J Clin Microbiol 40:2772–2778. doi:10.1128/JCM.40.8.2772-2778.2002 12149328 PMC120616

[14] Hay ID, Wang Y, Moradali MF, Rehman ZU, Rehm BHA. 2014. Genetics and regulation of bacterial alginate production. Environ Microbiol 16:2997–3011. doi:10.1111/1462-2920.12389 24428834

[15] Yu H, Boucher JC, Hibler NS, Deretic V. 1996. Virulence properties of Pseudomonas aeruginosa lacking the extreme-stress sigma factor AlgU (sigmaE). Infect Immun 64:2774–2781. doi:10.1128/iai.64.7.2774-2781.1996 8698507 PMC174138

[16] Cezairliyan BO, Sauer RT. 2009. Control of Pseudomonas aeruginosa AlgW protease cleavage of MucA by peptide signals and MucB. Mol Microbiol 72:368–379. doi:10.1111/j.1365-2958.2009.06654.x 19298369 PMC2754073

[17] Li S, Lou X, Xu Y, Teng X, Liu R, Zhang Q, Wu W, Wang Y, Bartlam M. 2019. Structural basis for the recognition of MucA by MucB and AlgU in Pseudomonas aeruginosa. FEBS J 286:4982–4994. doi:10.1111/febs.14995 31297938

[18] Joshi KK, Bergé M, Radhakrishnan SK, Viollier PH, Chien P. 2015. An adaptor hierarchy regulates proteolysis during a bacterial cell cycle. Cell 163:419–431. doi:10.1016/j.cell.2015.09.030 26451486 PMC4600535

[19] Olivares AO, Baker TA, Sauer RT. 2016. Mechanistic insights into bacterial AAA+ proteases and protein-remodelling machines. Nat Rev Microbiol 14:33–44. doi:10.1038/nrmicro.2015.4 26639779 PMC5458636

[20] Bolon DN, Grant RA, Baker TA, Sauer RT. 2004. Nucleotide-dependent substrate handoff from the SspB adaptor to the AAA+ ClpXP protease. Mol Cell 16:343–350. doi:10.1016/j.molcel.2004.10.001 15525508

[21] Ghanbarpour A, Fei X, Baker TA, Davis JH, Sauer RT. 2023. The SspB adaptor drives structural changes in the AAA+ ClpXP protease during ssrA-tagged substrate delivery. Proc Natl Acad Sci USA 120:e2219044120. doi:10.1073/pnas.2219044120 36730206 PMC9963277

[22] Flynn JM, Levchenko I, Sauer RT, Baker TA. 2004. Modulating substrate choice: the SspB adaptor delivers a regulator of the extracytoplasmic-stress response to the AAA+ protease ClpXP for degradation. Genes Dev 18:2292–2301. doi:10.1101/gad.1240104 15371343 PMC517522

[23] Kreda SM, Davis CW, Rose MC. 2012. CFTR, mucins, and mucus obstruction in cystic fibrosis. Cold Spring Harb Perspect Med 2:a009589. doi:10.1101/cshperspect.a009589 22951447 PMC3426818

[24] Turcios NL. 2020. Cystic fibrosis lung disease: an overview. Respir Care 65:233–251. doi:10.4187/respcare.06697 31772069

[25] Damron FH, Yu HD. 2011. Pseudomonas aeruginosa MucD regulates the alginate pathway through activation of MucA degradation via MucP proteolytic activity. J Bacteriol 193:286–291. doi:10.1128/JB.01132-10 21036998 PMC3019965

[26] Pedersen SS, Høiby N, Espersen F, Koch C. 1992. Role of alginate in infection with mucoid Pseudomonas aeruginosa in cystic fibrosis. Thorax 47:6–13. doi:10.1136/thx.47.1.6 1539148 PMC463537

[27] Malhotra S, Hayes D, Wozniak DJ. 2019. Cystic fibrosis and Pseudomonas aeruginosa: the host-microbe interface. Clin Microbiol Rev 32:e00138-18. doi:10.1128/CMR.00138-18 31142499 PMC6589863

[28] Kong W, Zhao J, Kang H, Zhu M, Zhou T, Deng X, Liang H. 2015. ChIP-seq reveals the global regulator AlgR mediating cyclic di-GMP synthesis in Pseudomonas aeruginosa. Nucleic Acids Res 43:8268–8282. doi:10.1093/nar/gkv747 26206672 PMC4787818

[29] Ramsey DM, Wozniak DJ. 2005. Understanding the control of Pseudomonas aeruginosa alginate synthesis and the prospects for management of chronic infections in cystic fibrosis. Mol Microbiol 56:309–322. doi:10.1111/j.1365-2958.2005.04552.x 15813726

[30] Wu W, Badrane H, Arora S, Baker HV, Jin S. 2004. MucA-mediated coordination of type III secretion and alginate synthesis in Pseudomonas aeruginosa. J Bacteriol 186:7575–7585. doi:10.1128/JB.186.22.7575-7585.2004 15516570 PMC524895

[31] Jones AK, Fulcher NB, Balzer GJ, Urbanowski ML, Pritchett CL, Schurr MJ, Yahr TL, Wolfgang MC. 2010. Activation of the Pseudomonas aeruginosa AlgU regulon through mucA mutation inhibits cyclic AMP/Vfr signaling. J Bacteriol 192:5709–5717. doi:10.1128/JB.00526-10 20817772 PMC2953679

[32] Qiu D, Eisinger VM, Rowen DW, Yu HD. 2007. Regulated proteolysis controls mucoid conversion in Pseudomonas aeruginosa. Proc Natl Acad Sci USA 104:8107–8112. doi:10.1073/pnas.0702660104 17470813 PMC1876579

[33] Qiu D, Eisinger VM, Head NE, Pier GB, Yu HD. 2008. ClpXP proteases positively regulate alginate overexpression and mucoid conversion in Pseudomonas aeruginosa. Microbiology (Reading) 154:2119–2130. doi:10.1099/mic.0.2008/017368-0 18599839 PMC2995304

[34] Yin Y, Withers TR, Wang X, Yu HD. 2013. Evidence for sigma factor competition in the regulation of alginate production by Pseudomonas aeruginosa. PLoS One 8:e72329. doi:10.1371/journal.pone.0072329 23991093 PMC3750012

[35] Yu Z, Zhang J, Ding M, Wu S, Zhang M, Yin J, Meng Q, Shuangjia Li. 2020. SspA positively controls exopolysaccharides production and biofilm formation by up-regulating the algU expression in Pseudoalteromonas sp. R3. Biochem Biophys Res Commun 533:988–994. doi:10.1016/j.bbrc.2020.09.118 33010891

[36] Hmelo LR, Borlee BR, Almblad H, Love ME, Randall TE, Tseng BS, Lin C, Irie Y, Storek KM, Yang JJ, Siehnel RJ, Howell PL, Singh PK, Tolker-Nielsen T, Parsek MR, Schweizer HP, Harrison JJ. 2015. Precision-engineering the Pseudomonas aeruginosa genome with two-step allelic exchange. Nat Protoc 10:1820–1841. doi:10.1038/nprot.2015.115 26492139 PMC4862005

[37] Liu L, Li T, Cheng X-J, Peng C-T, Li C-C, He L-H, Ju S-M, Wang N-Y, Ye T-H, Lian M, Xiao Q-J, Song Y-J, Zhu Y-B, Yu L-T, Wang Z-L, Bao R. 2018. Structural and functional studies on Pseudomonas aeruginosa DspI: implications for its role in DSF biosynthesis. Sci Rep 8:3928. doi:10.1038/s41598-018-22300-1 29500457 PMC5834635

[38] Lin J, Zhang W, Cheng J, Yang X, Zhu K, Wang Y, Wei G, Qian P-Y, Luo Z-Q, Shen X. 2017. A Pseudomonas T6SS effector recruits PQS-containing outer membrane vesicles for iron acquisition. Nat Commun 8:14888. doi:10.1038/ncomms14888 28348410 PMC5379069

[39] Deng J, Wu Y, Zheng Z, Chen N, Luo X, Tang H, Keasling JD. 2021. A synthetic promoter system for well-controlled protein expression with different carbon sources in Saccharomyces cerevisiae. Microb Cell Fact 20:202. doi:10.1186/s12934-021-01691-3 34663323 PMC8522093

[40] Rehman ZU, Rehm BHA. 2013. Dual roles of Pseudomonas aeruginosa AlgE in secretion of the virulence factor alginate and formation of the secretion complex. Appl Environ Microbiol 79:2002–2011. doi:10.1128/AEM.03960-12 23335756 PMC3592226

[41] Jain S, Ohman DE. 1998. Deletion of algK in mucoid Pseudomonas aeruginosa blocks alginate polymer formation and results in uronic acid secretion. J Bacteriol 180:634–641. doi:10.1128/JB.180.3.634-641.1998 9457868 PMC106932

[42] Gennarelli M, Lucarelli M, Capon F, Pizzuti A, Merlini L, Angelini C, Novelli G, Dallapiccola B. 1995. Survival motor-neuron gene transcript analysis in muscles from spinal muscular-atrophy patients. Biochem Biophys Res Commun 213:342–348. doi:10.1006/bbrc.1995.2135 7639755

[43] Lefebvre S, Burlet P, Liu Q, Bertrandy S, Clermont O, Munnich A, Dreyfuss G, Melki J. 1997. Correlation between severity and SMN protein level in spinal muscular atrophy. Nat Genet 16:265–269. doi:10.1038/ng0797-265 9207792

[44] Peng C-T, Liu L, Li C-C, He L-H, Li T, Shen Y-L, Gao C, Wang N-Y, Xia Y, Zhu Y-B, Song Y-J, Lei Q, Yu L-T, Bao R. 2017. Structure–function relationship of aminopeptidase P from Pseudomonas aeruginosa. Front Microbiol 8:2385. doi:10.3389/fmicb.2017.02385 29259588 PMC5723419

