# Supplementary material for: Structural basis of the AlgU-MucAcyto interaction and SspB-mediated degradation in Pseudomonas aeruginosa stress response
Source: mBio. 2026 May 19;17(6):e00148-26. doi: 10.1128/mbio.00148-26 (PMC13251409; doi:10.1128/mbio.00148-26)
Supplement: Supplemental Figures — Fig. S1 to S9. [file mbio.00148-26-s0001.docx]

**Supplementary Materials​**

**Structural Basis of the AlgU-MucA^cyto^ Interaction and SspB-Mediated Degradation in *Pseudomonas aeruginosa* Stress Response​**

Tao Li^1#^, Yingzhi Wang^4#^, Ninglin Zhao^2#^, Chunlei Ge^1^, Cuiling Wu^1^, Ke Li^1^, Li Li^1^, Zhiqiang Wang^5^, Ying Chen^1^, Zhenpu Chen^1^, Weike Li^1^, Yang Liu^1^, Zhonghui Wang^1^, Yun Sha^1^, Hong Yao^1^, Yibo Zhu^2,3^*, Rui Bao^2^*

^1^ Cancer Biotherapy Center & Cancer Research Institute, Yunnan Cancer Hospital, The Third Affiliated Hospital of Kunming Medical University, Peking University Cancer Hospital Yunnan, Kunming, China.

^2^ Center of Infectious Diseases, Division of Infectious Diseases in State Key Laboratory of Biotherapy, West China Hospital, Sichuan University, Chengdu, Sichuan, 610041, China.

^3^ Accurate Biotechnology (Hunan) Co., Ltd, Changsha 410006, China.

^4^ University-Town Hospital of Chongqing Medical University, Chongqing, China.

^5^ Department of Radiation Oncology, First Affiliated Hospital of Kunming Medical University, Kunming 650032, Yunnan, China.

***Corresponding author**: Rui Bao, [baorui@scu.edu.cn](mailto:baorui@scu.edu.cn).

^#^Authors contributed equally to this work. Tao Li, litaove@163.com.


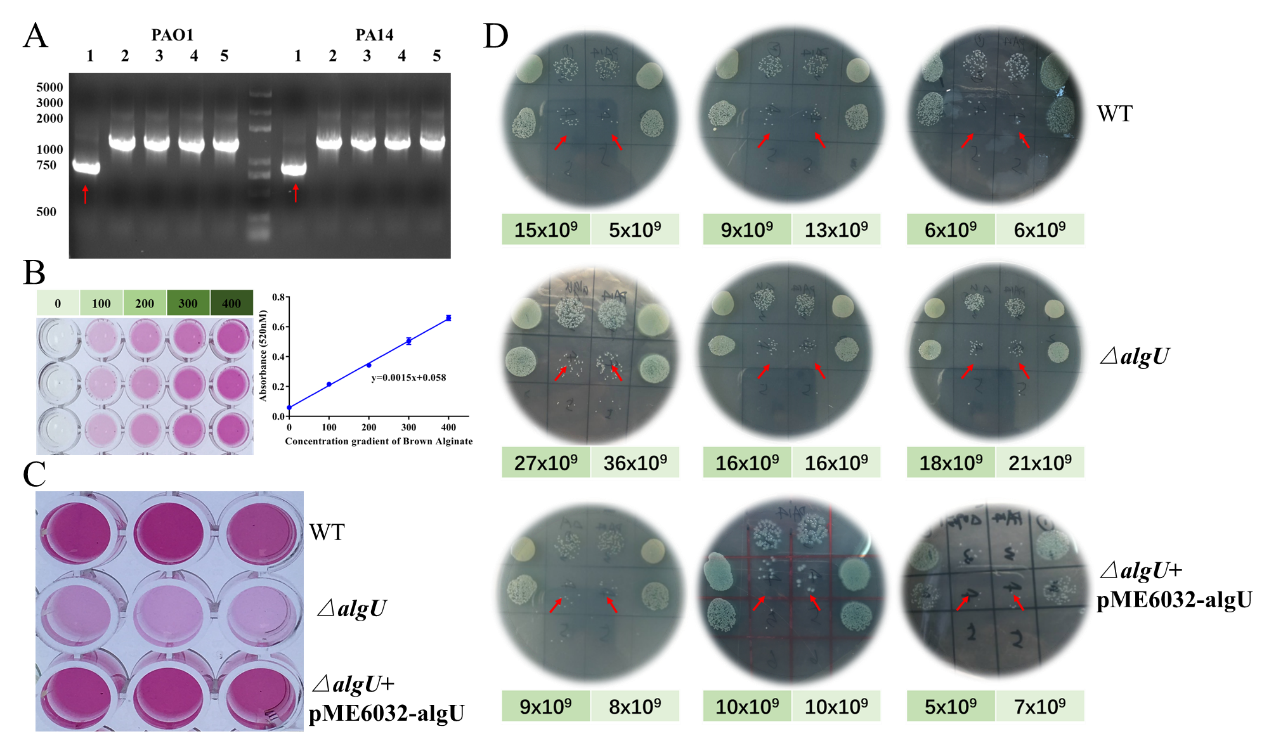


**Figure S1. Original data for the construction of *ΔalgU* strain, alginate determination and CFU counting.** (A). Construction of strain *ΔalgU* by two-step allelic exchange strategy. The strain was screened by PCR and sequencing. WT: wild-type. (B) Standard Curve for Alginate Quantification​. Commercial alginate samples of varying concentrations (mg/mL) were mixed with a solution of borate-sulfuric acid and the carbazole reagent. Following this, the mixture was subjected to heating at a temperature of 55 °C for a duration of 30 minutes. Post-heating, the absorbance of the solution was measured at A_520_. The alginate content within the cell culture was quantified and reported in terms of micrograms of alginate per milligram of cell weight. (C) Alginate produced by *P. aeruginosa* PAO1 and its variants. (D) Cell cloning was conducted to analyze the cell contents of Pseudomonas aeruginosa in growth dishes, which had been previously infected with A549 cells by various strains.


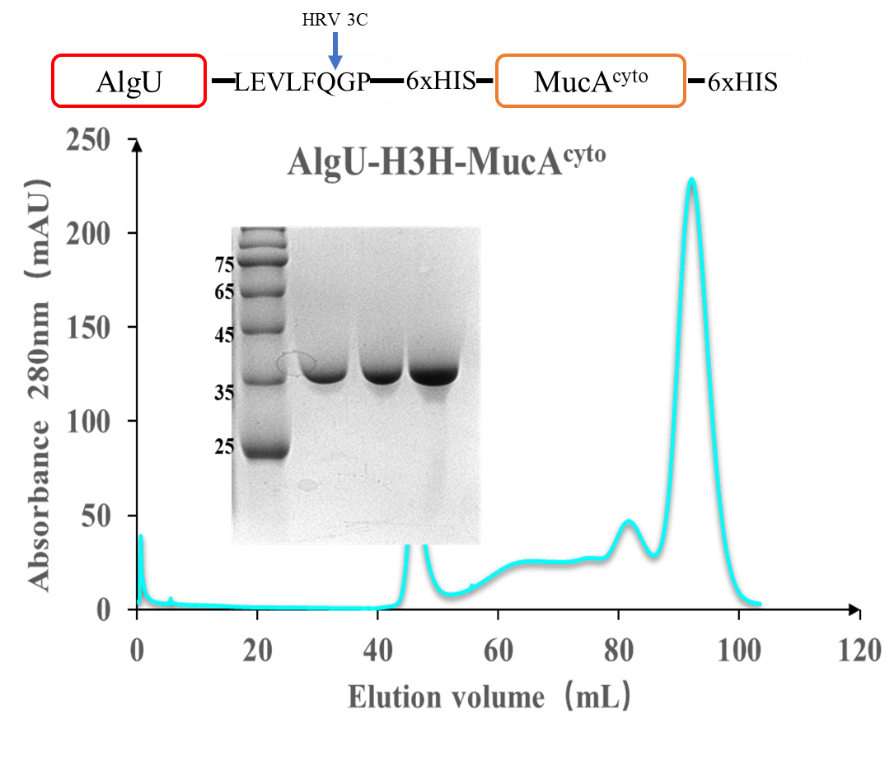


**Figure S2. Design and purification strategy for the AlgU-H3H-MucA^cyto^ fusion protein.​​** Schematic representation of the engineered construct: AlgU and MucA^cyto^ domains are connected by a bifunctional linker encoding an HRV 3C protease recognition site (LEVLFQ↓GP) and an N-terminal hexahistidine affinity tag (H3H). This fusion strategy enabled high-yield expression and purification of the soluble AlgU-H3H-MucA^cyto^ complex prior to proteolytic cleavage.


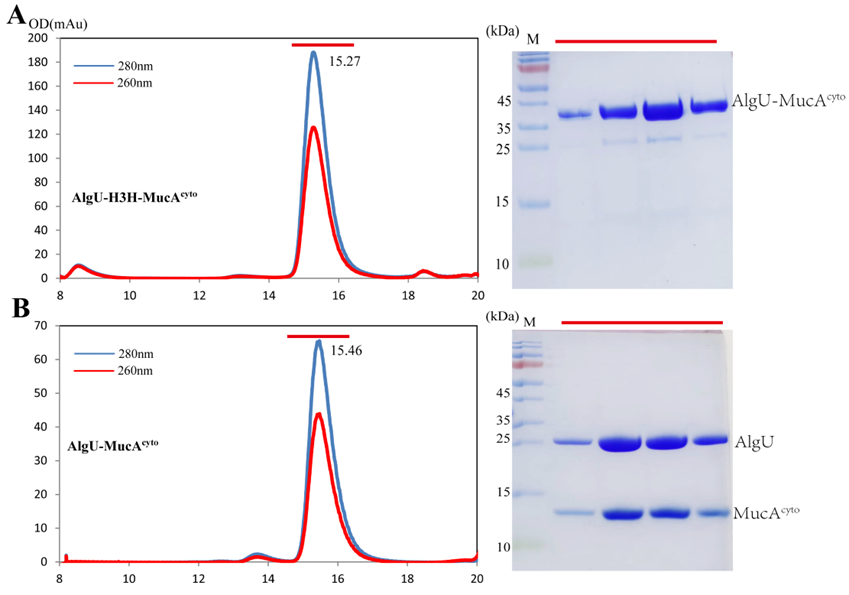


**Figure S3. Size-exclusion chromatography analysis of AlgU-H3H-MucA^cyto^ fusion protein before and after HRV 3C protease cleavage.​​** (Left) Elution profiles of the intact fusion protein and post-digestion complex on a Superdex 200 Increase 10/300 GL column. (Right) SDS-PAGE analysis of corresponding SEC fractions.


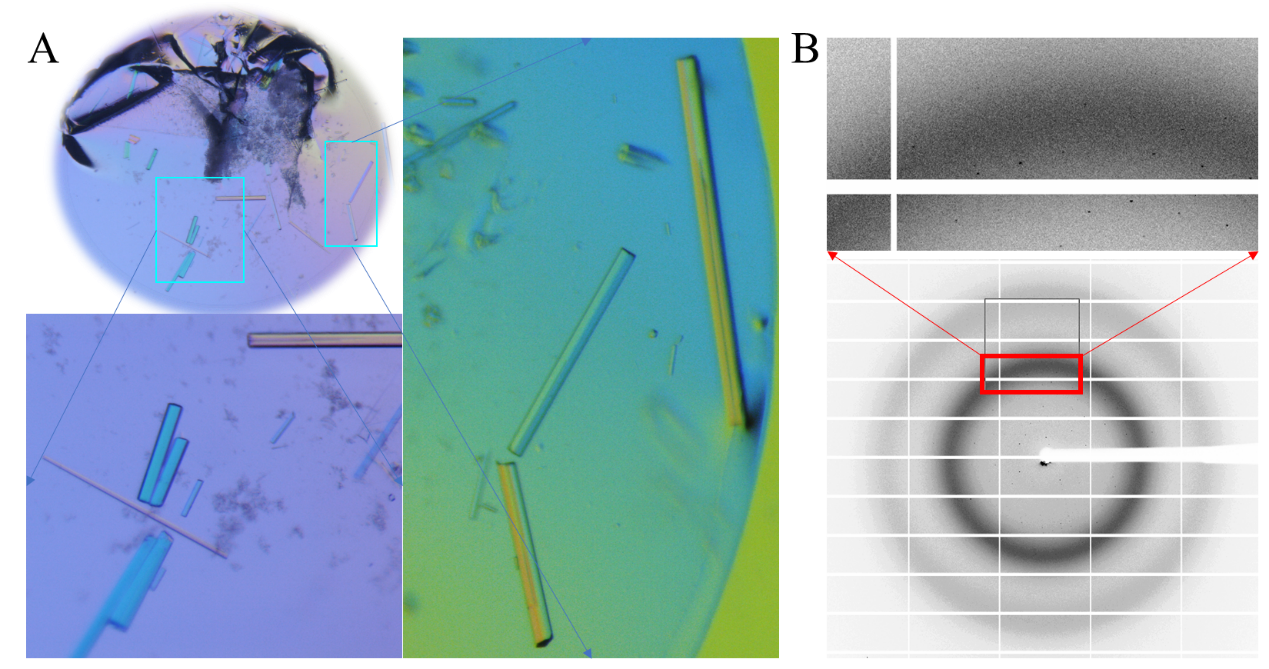


**Figure S4. Crystal of AlgU-MucA^cyto^ and crystallography data.** (A). AlgU-MucA^cyto^ crystal growth in PEG F6: 10% 2-Propanol 0.1M BICINE ph8.5(NаOH), 30% PEG1500. (B). X-ray diffraction of AlgU-MucA^cyto^.


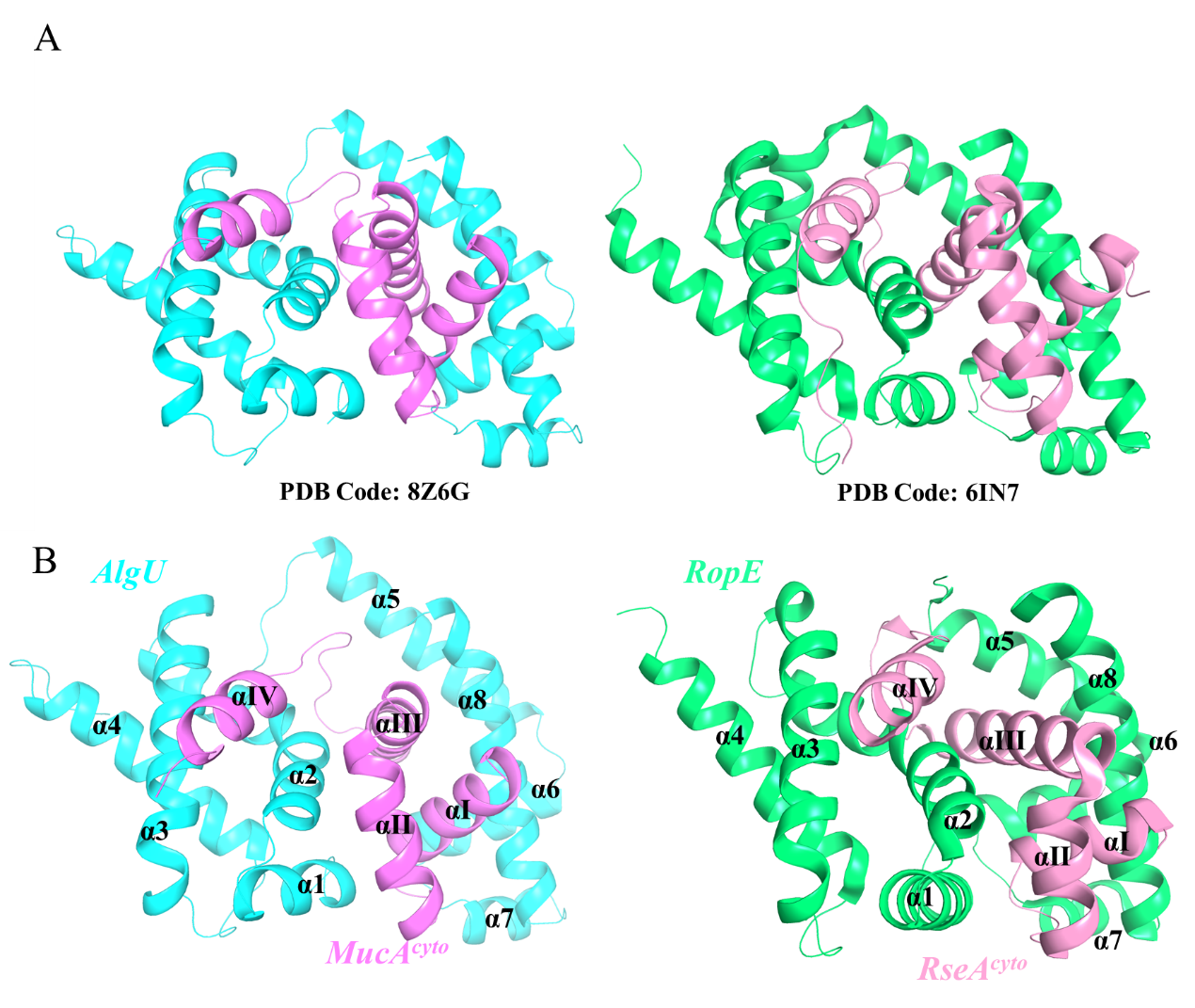


**Figure S5. The overall structure and refinement data of the AlgU-MucA^cyto^.** Overall structure alignment of complex AlgU-MucA^cyto^ with identified 6IN7 (A) and RopE-RseA^cyto^ complexes (PDB code: 1OR7 in *E. coli* (B).


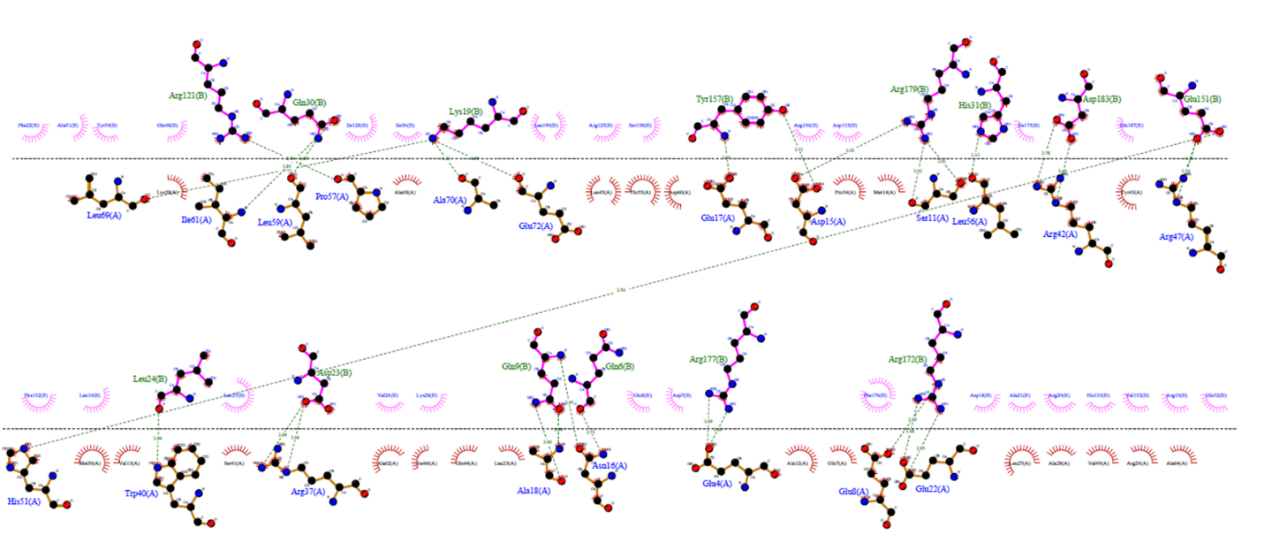


Figure S6. The interaction between AlgU and MucA^cyto^ was generated by Ligplus software.


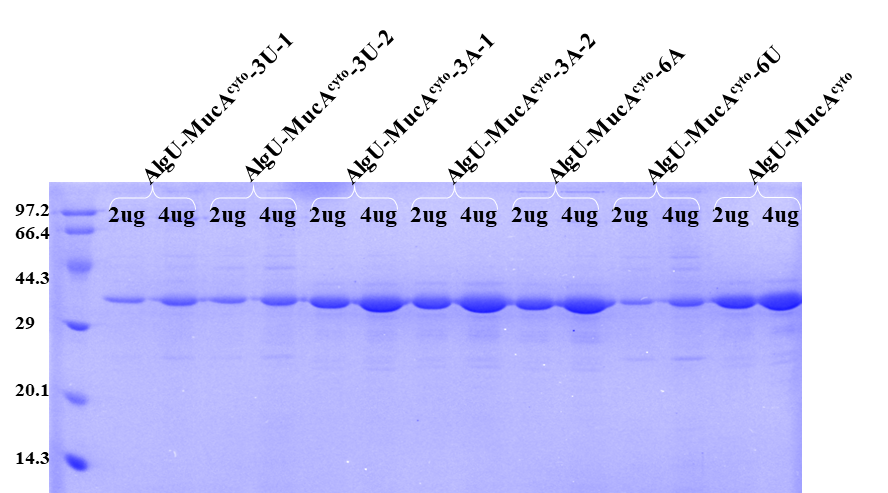


**Figure S7. SDS-PAGE analysis of purified mutant AlgU-H3H-MucA^cyto^ fusion variants​.** All constructs maintained intact migration profiles at expected molecular weight (~35 kDa), confirming successful purification of soluble proteins. Triple-cluster MucA^cyto^-targeted mutants AlgU-MucA^cyto^-3A-1 (E8A/E17A/E22A) and AlgU-MucAcyto-3A-2 (R37A/R42A/R47A), and AlgU-targeted mutants AlgU-MucA^cyto^-3U-1 (R150A/E151A/Y157A) and AlgU-MucA^cyto^-3U-2 (R172A/R177A/R179A). Six-site mutants: MucA^cyto^-targeted AlgU-MucA^cyto^-6A (E8A/E17A/E22A/R37A/R42A/R47A) and AlgU-targeted AlgU-MucA^cyto^-6U (R150A/E151A/Y157A/R172A/R177A/R179A).


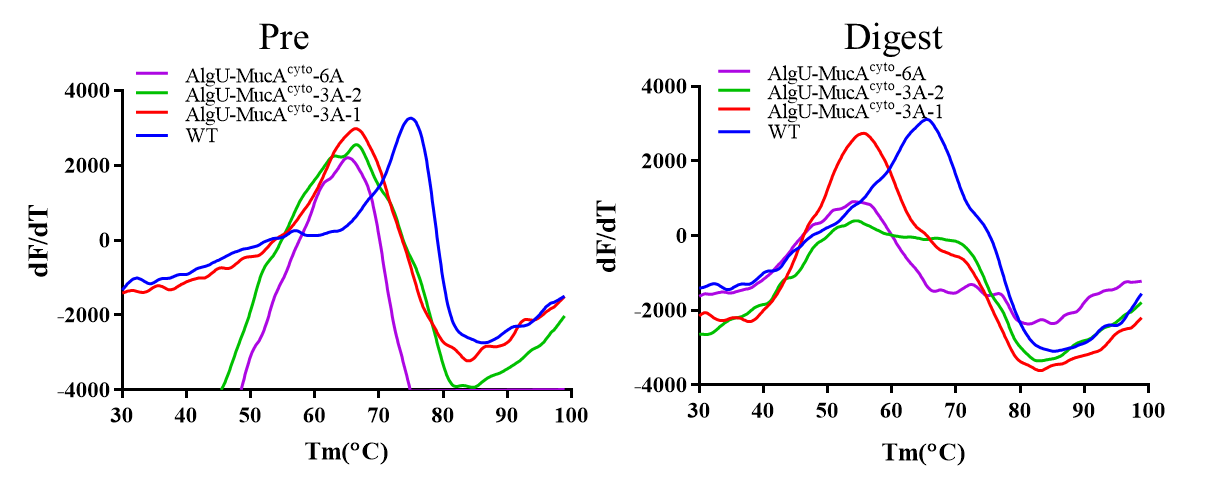


**Figure S8.** DSF identification of the thermal denaturation curves for AlgU-MucA^cyto^, Triple-cluster MucA^cyto^-targeted mutants AlgU-MucA^cyto^-3A-1 (E8A/E17A/E22A) and AlgU-MucA^cyto^-6A (E8A/E17A/E22A/R37A/R42A/R47A).


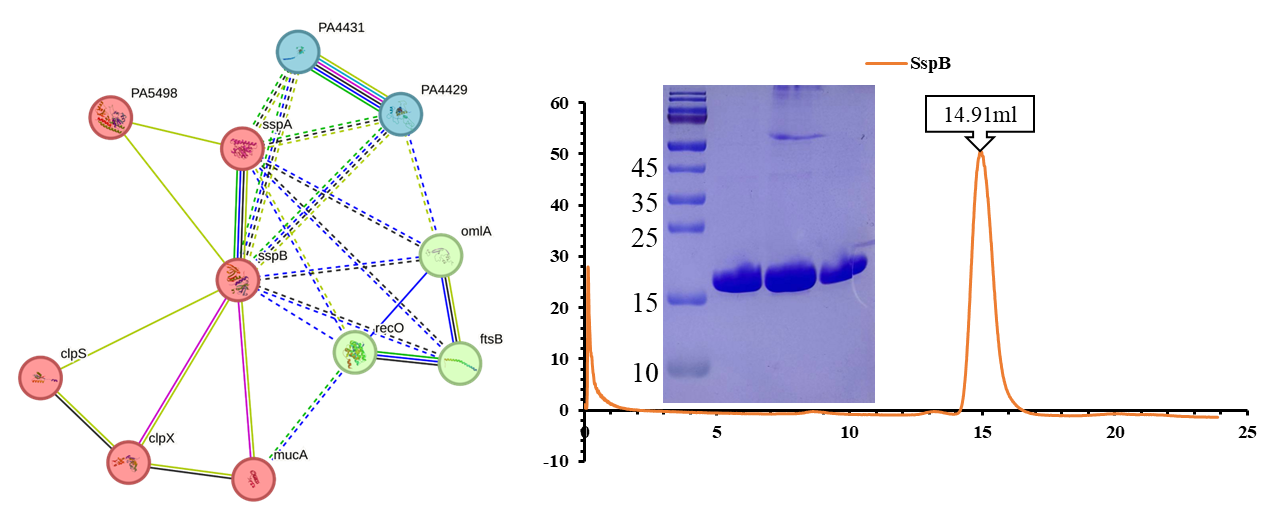


**Figure S9.** **Prediction and purification of SspB-interacting proteins.​​** (A) STRING database analysis of potential SspB interactors. (B) SEC elution profile of purified His₆-tagged SspB (Superdex 200 Increase 10/300 GL column).
